# Investigating cytosolic 5′-nucleotidase II family genes as candidates for neuropsychiatric disorders in *Drosophila* (114/150 chr)

**DOI:** 10.1038/s41398-020-01149-x

**Published:** 2021-01-18

**Authors:** Euginia L. Singgih, Monique van der Voet, Marlies Schimmel-Naber, Emma L. Brinkmann, Annette Schenck, Barbara Franke

**Affiliations:** 1 Department of Human Genetics, Donders Institute for Brain, Cognition and Behaviour, Radboud University Medical Center, Nijmegen, The Netherlands; 2 Department of Psychiatry, Donders Institute for Brain, Cognition and Behaviour, Radboud University Medical Center, Nijmegen, The Netherlands

**Keywords:** Molecular neuroscience, ADHD, Schizophrenia

## Abstract

Cytosolic 5′-nucleotidases II (cNT5-II) are an evolutionary conserved family of 5′-nucleotidases that catalyze the intracellular hydrolysis of nucleotides. In humans, the family is encoded by five genes, namely *NT5C2*, *NT5DC1*, *NT5DC2*, *NT5DC3*, and *NT5DC4*. While very little is known about the role of these genes in the nervous system, several of them have been associated with neuropsychiatric disorders. Here, we tested whether manipulating neuronal expression of cNT5-II orthologues affects neuropsychiatric disorders-related phenotypes in the model organism *Drosophila melanogaster*. We investigated the brain expression of *Drosophila* orthologues of cNT5-II family (*dNT5A*-*CG2277*, *dNT5B*-*CG32549*, and *dNT5C-CG1814*) using quantitative real-time polymerase chain reaction (qRT-PCR). Using the *UAS*/*Gal4* system, we also manipulated the expression of these genes specifically in neurons. The knockdown was subjected to neuropsychiatric disorder-relevant behavioral assays, namely light-off jump reflex habituation and locomotor activity, and sleep was measured. In addition, neuromuscular junction synaptic morphology was assessed. We found that *dNT5A*, *dNT5B*, and *dNT5C* were all expressed in the brain. *dNT5C* was particularly enriched in the brain, especially at pharate and adult stages. Pan-neuronal knockdown of *dNT5A* and *dNT5C* showed impaired habituation learning. Knockdown of each of the genes also consistently led to mildly reduced activity and/or increased sleep. None of the knockdown models displayed significant alterations in synaptic morphology. In conclusion, in addition to genetic associations with psychiatric disorders in humans, altered expression of cNT5-II genes in the *Drosophila* nervous system plays a role in disease-relevant behaviors.

## Introduction

The family of cytosolic 5′-nucleotidase II (cNT5-II) consists of five highly conserved enzymes, encoded by the *NT5C2*, *NT5DC1*, *NT5DC2*, *NT5DC3*, and *NT5DC4* genes. Research so far has mainly concentrated on NT5C2, which catalyzes the dephosphorylation of purine nucleotides into corresponding purine nucleosides. The enzyme has a high affinity for 5′-inosine monophosphate (IMP) and 5′-guanosine monophosphate (GMP) and is likely to play a role in regulating cellular IMP and GMP levels^1^. NT5C2 has also been shown to negatively regulate phosphorylation of the alpha subunit of 5′-adenosine monophosphate-activated protein kinase (AMPK alpha) and protein translation^2,3^. No research has yet addressed the 5′-nucleotidase activity of other family members; however, domain similarity infers conserved catalytic activity.

Dysfunction of members of the cNT5-II family has been linked to immunological disorders, sensitivity to cancer treatment, and to metabolic disorders^4^. Additionally, associations with neurological and psychiatric disorders have been reported (Supplementary Table 1A–E). Truncation and aberrant splicing of *NT5C2* causes a form of spastic paraplegia (SPG45), which is frequently accompanied by intellectual disability (ID), a thin corpus callosum, and symptoms of attention-deficit/hyperactivity disorder (ADHD)^5–8^. Neuronal expression of *Drosophila NT5C2* orthologue has also been shown to be essential for locomotor performance^2^. Associations of *NT5C2* have additionally been seen with cognitive abilities as well as schizophrenia in several genome-wide association studies (GWASs)^9^ (Supplementary Table 1A). The schizophrenia risk variants rs11191419 and ch10_104957618_I were associated with reduced *NT5C2* expression in the fetal and adult brain^10^. Furthermore, a locus containing *NT5C2* was genome-wide significantly associated with insomnia in a recent GWAS^11^. Another family member, *NT5DC2*, has also repeatedly been associated with cognitive performance, schizophrenia, and bipolar disorder^9^ (Supplementary Table 1C). It was shown that *NT5DC2* can competitively inhibit monoamine synthesis by inhibiting tyrosine hydroxylase^12^. Suggestive associations with ADHD and bipolar disorder have also been noted for *NT5DC1* in genetic association studies^13,14^, and the expression level of *Nt5dc3* in a mouse model has been positively correlated with reversal learning performance^15^. No links with brain function have been reported for *NT5DC4* so far.

Little is yet known about how the cNT5-II enzymes affect the brain and nervous system. Animal models provide excellent opportunities to deepen our understanding of this highly conserved gene family. The fruit fly *Drosophila melanogaster* is a well-established, cost-efficient and time-efficient model to investigate human traits and diseases, both somatic and brain-based. Despite the evolutionary distance between flies and humans, a strong conservation of genes and regulatory networks exists (e.g., see refs. ^16,17^), and nearly 75% of disease-related human genes have functional *Drosophila* orthologs^18,19^. Genetic manipulation is fast and easy, e.g., through the *UAS*/*Gal4* system, which allows manipulation of gene expression specifically in desired tissues or cell populations, such as neurons^20^. We have previously shown that manipulation of ADHD genes in *Drosophila* caused altered locomotor activity and reduced sleep^21,22^. Additionally, in a large-scale screen of *Drosophila* models of neurodevelopmental and psychiatric disorders, we demonstrated that deficits in habituation learning, a simple form of learning that serves as a cognitive filter, were highly abundant^23^. Altered habituation has been reported in, for example, Autism Spectrum Disorder (ASD), ADHD and schizophrenia^24^. Here, we studied the expression of cNT5-II genes, i.e., *CG1814*, *CG2277*, and *CG32549* (Supplementary Fig. 1), in the nervous system of *Drosophila* across the lifespan and investigated effects of neuronal knockdown of the *Drosophila* orthologues on neuropsychiatric disorder-relevant phenotypes of locomotor activity, sleep, habituation, as well as on synapse morphology.

## Materials and methods

### Fly stocks

Flies were maintained and crossed on standard corn meal medium at 28 °C, 60% relative humidity, unless specified. The following inducible *UAS*-RNAi *Drosophila* lines were obtained from the Vienna *Drosophila* Resource Center (VDRC), RNAi_1_–v19096 (*w*^*1118*^;; *P[GD8619]v19096*) and RNAi_2_–v106195 (*P[KK102549]VIE-260B*/CyO) to knockdown *CG1814* (termed *dNT5C*); RNAi_1_–v20869 (*w*^*1118*^;; *P[GD9772]v20869*) and RNAi_2_–v108903 (*P[KK105724]VIE-260B*) to knockdown *CG2277* (termed *dNT5A*); RNAi_1_–v30079 (*w*^*1118*^;; *P[GD14594]v30079*) and RNAi_2_–v103916 (*P[KK101772]VIE-260B*) to knockdown *CG32549* (termed *dNT5B*)^25^. The v60000 line served as genetic background control for RNAi_1_ and v60100 for RNAi_2_. Validation experiments of the RNAi lines are available in Supplementary Fig. 2. The v110662 line (*P[KK100579]VIE-260B*), contains insertion at both 30B and 40D landing sites, and v60101, containing a *UAS* sequence at the 40D landing site, served as positive controls for diagnostic PCR determining the landing site. For expression level analysis, wild type flies derived from Canton S and Oregon R strains were used. The following drivers were used to induce knockdown ubiquitously (w*;; *da.G32*-*Gal4*) or pan-neuronally (yw* UAS-*Dcr2 hs*(X);; *nSyb*-*Gal4*). *UAS*-*Dcr2* was used to increase the efficiency of the knockdown^25^. For the habituation assay, two copies of *GMR*-*w*^*IR*^ element were included in the driver line to suppress the eye color in the progeny, which is required for the light-off jump reflex (LOJR) to occur (*w*^*-*^; *2xGMR*-*w*^*IR*^; *nSyb*-*Gal4*, *UAS*-*Dcr2*)^26^.

### RNA extraction and quantitative real time PCR (qRT-PCR)

To determine *Drosophila* cNT5-II (*dNT5*) genes expression in the brain, ten male animals of each indicated developmental stage were collected, washed from excess food and dissected in ice-cold phosphate buffered saline (PBS) to dissect the brains from the rest of the body. Both tissue fractions were submerged in RNAlater (Sigma, Germany) and snap-frozen in liquid nitrogen. The investigated developmental stage were chosen based on the ones represented in data resources such as FlyAtlas and modENCODE^27,28^. The total RNA was then isolated with Arcturus Picopure (Thermo Fisher Scientific, Lithuania) and measured with Qubit HS RNA assay (Thermo Fisher Scientific, Lithuania) or Nanodrop (Thermo Fisher Scientific). Complementary DNA (cDNA) was synthesized from total RNA with iScript kit (BioRad, Hercules, CA). The cDNA was diluted 10× and subjected to qRT-PCR using Power SYBR® Green PCR master mix (Thermo Fisher Scientific, UK) with 7900HT Fast Real-Time PCR system (Applied Biosystems) in 384-well plate format (primer sequences available in Supplementary Table 2). Primer sets were designed with Primer3^29^, unless specified. *eIF-1A* and *αTub84B* were used as internal control. Cycle threshold (*C*_T_) values were determined with SDS 2.4 (Applied Biosystems) and the difference of expression level was determined using ΔΔ*C*_T_ method^30^. Significance was calculated using paired *T*-test with Prism 5.03 (Graphpad Software, San Diego, CA).

### Neuromuscular junction visualization and quantification

Neuromuscular junction visualization and quantification were performed as described by Nijhof et al. and Castels-Nobau et al.^31,32^. In brief, third instar wandering male larvae were dissected according to the open book preparation method and preserved in 3.7% paraformaldehyde for 30 min. The larvae were incubated overnight at 4 °C with mouse anti-nc82 (1:125) (Developmental Studies Hybridoma Bank), followed by 2 h incubation at room temperature with Alexa 488 goat-anti-mouse (1:125) (Invitrogen Molecular Probes, Eugene, OR, USA) to visualize active zones. To visualize the postsynaptic morphology of the motor neuron terminals, specimen were further incubated for 1.5 h at room temperature with anti-Dlg-1 antibody (1:25) (Developmental Studies Hybridoma Bank) that had previously been conjugated with the Zenon Alexa Fluor 568 Mouse IgG1 labeling kit (Invitrogen, CA, USA). The immunolabeled larvae were mounted in Vectashield (Vector Laboratories, Burlingame, CA, USA) and imaged with a Zeiss Axio Imager microscope at 630x magnification using 63× oil immersion objective lens with ApoTome.2 (Zeiss). Images were analyzed with the *Drosophila* NMJ morphometrics macro^31,32^ in FIJI 1.49k^33^ to obtain eight interdependent morphology-related parameters (area, perimeter, length, longest branch length, bouton count, branch point, branch number, and island) and number of active zone. For each genotype, synapses from 5 to 10 animals were analyzed. One-way analysis of variance (ANOVA) with Dunnett’s post-hoc test against appropriate genetic background controls was performed with Prism 5.03 (GraphPad Software, San Diego, CA, USA) to obtain an adjusted *p*-value (*p*_adj_).

### Behavioral assays

#### Habituation

The habituation assay was performed as previously described^23^. Flies were grown and maintained at 25 °C, 70% humidity for habituation experiments. Male flies were collected with CO_2_, allowed to recover for at least 48 h and tested in the light-off jump reflex system (Aktogen, Hungary) at 3–7 days post-eclosion. Individually housed flies received 100 light-off pulses for 15 ms with 1 s interpulse interval. The frequency of wing vibration following a jump response was measured at each trial and a threshold was applied to filter out background noise. Data were collected and analyzed in a custom made Labview Software (National Instruments). Only genotypes with >50% of flies that jump at least once within the first five trials (*n* jumpers) were analyzed. The total number of tested flies (*n* total) and the number of flies jumping on the first five trials (*n* jumpers) are provided in Supplementary Table 3. Habituation was quantified using the trials-to-criterion (TTC), which corresponds to the number of the trial at which a fly stops jumping for at least five consecutive trials. General linear model regression analysis with correction according to the number of RNAi lines tested was performed on the log-TTC values using R statistical software to obtain an adjusted *p*-value (*p*_adj_).

#### Activity and sleep monitoring

Activity and sleep monitoring were performed as described by van der Voet et al. and Klein et al.^21,22^. Male flies age 3–5 days were collected with CO_2_ and allowed to recover for at least 24 h. The flies were then monitored using *Drosophila* activity monitoring (DAM) system (Trikinetics, Waltham, MA, USA) at 28 °C 60% relative humidity for 4 days in 12:12-h light:dark scheme. Activity counts were collected in 30-s bins. Activity and sleep analysis were performed with Sleep and Circadian Analysis MATLAB Program (SCAMP)^34^; sleep was defined as a minimum of 5 min of inactivity^35^. Both activity and sleep were averaged over 4 days and plotted in 30-min bins. Day was defined as the interval between zeitgeber (ZT) 0–12 h and night as ZT 12–24 h. Day and night total activity, total sleep, activity while awake, sleep latency, sleep bout, and sleep bout duration were averaged over 4 days and were separately tested with one-way ANOVA, followed by Dunnett’s post-hoc test against their respective genetic background controls in Prism 5.03 (GraphPad Software, San Diego, CA, USA) to obtain final *p*-value (*p*_adj_).

## Results

### Expression of cNT5-II genes in the *Drosophila* central nervous system across lifespan

**Fig. 1:**
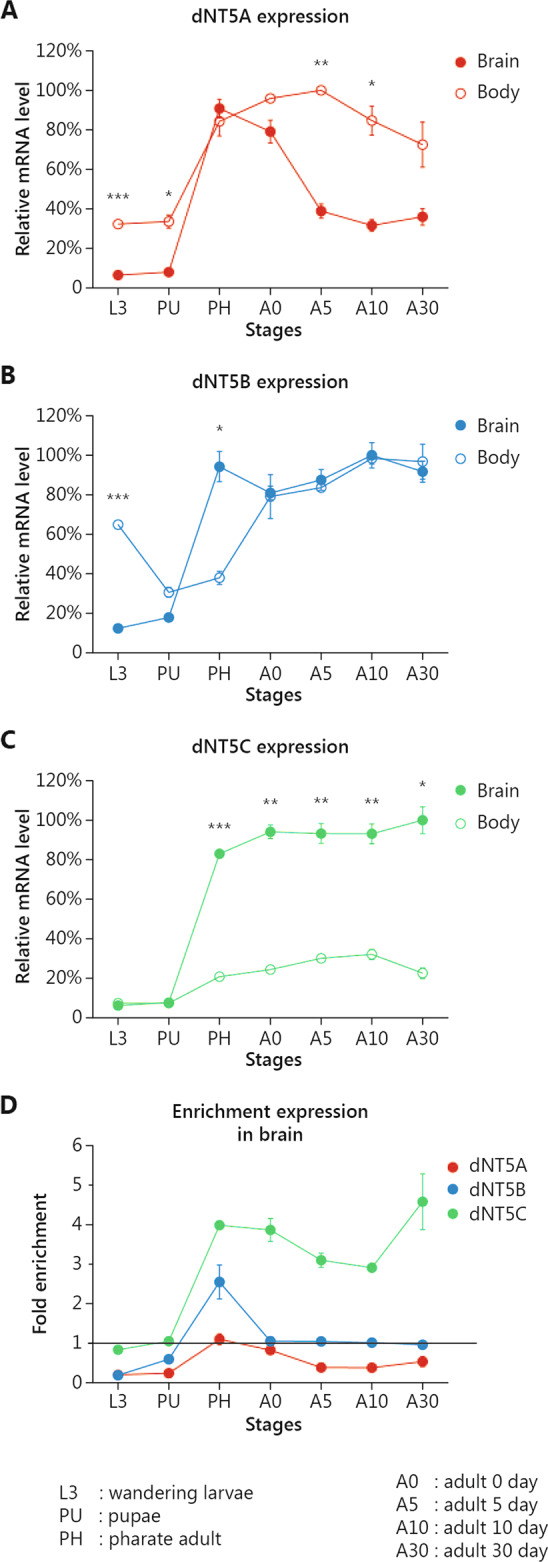
*dNT5* genes are expressed in the *Drosophila* brain, where *dNT5C* shows the highest enrichment compared to *dNT5A* and *dNT5B*. **A**–**C** mRNA levels of **A** *dNT5A*, **B** *dNT5B*, and **C** *dNT5C* in brain (filled circles) and rest of the body (empty circles) at different developmental stages, quantified by qRT-PCR relative to the maximum value detected among the assessed stages (100%). **D** Enrichment of expression levels in the brain compared to the body for each individual dNT5 gene. Error bars represent standard error of the mean. Paired *T*-test was performed to obtain a *p*-value. (*p < 0.05, **p < 0.01, ***p < 0.001). *N* = 3 biological replicates/stage, ten animals/replicate.

We investigated the following cNT5-II gene orthologues in *Drosophila*: *dNT5A* (*CG2277*, ortholog of *NT5DC1*), *dNT5B* (*CG32549*, ortholog of *NT5C2* and *NTDC4*), and *dNT5C* (*CG1814*, ortholog of *NT5DC2* and *NT5DC3*). The phylogenetic relationships between these genes are visualized in Supplementary Fig. 1. Investigating the expression of the *Drosophila* cNT5-II (*dNT5*) genes using qRT-PCR analysis to determine expression levels of each gene in the brain relative to the rest of the body across developmental stages, we found all three genes to be expressed in the brain (Fig. 1A–C). Expression of the genes was low at L3 larval (L3) and early pupal (PU) stages and increased from late pupal/pharate (PH) stage onwards. Expression of *dNT5A* was consistently lower in the brain than in the rest of the body, except for the PH stage (Fig. 1A, D). Also, *dNT5B* expression was initially higher in the rest of the body than in the brain at L3 and PU stages, but at the PH stage, brain expression increased up to 5-fold and remained similar to the expression in the body throughout adult stages (A0–A30) (Fig. 1B, D). Expression of *dNT5C* was initially similar in the brain and the rest of the body, but with onset at from the PH stage, brain expression became highly enriched and remained so throughout adult stages (Fig. 1C, D). Absolute expression levels, and thereby also absolute differences between expression of the three paralogous genes cannot be deduced from qRT-PCR data.

### Altered expression of dNT5 genes in RNAi-mediated knockdown

We validated available RNAi lines targeting each member of the dNT5 family. All RNAi constructs are predicted to be highly specific, with <0.5% predicted off-target 19-mers (s19 score >0.99, obtained from VDRC website, www.vdrc.at). First, we performed diagnostic PCR to map the integration site of *UAS*-RNAi_2_, as constructs from the VDRC KK collection might be inserted in chromosome 2 at either 30B and/or 40D locus. *dNT5A* RNAi_2_ and *dNT5C* RNAi_2_ constructs were integrated only at the 30B locus, and *dNT5B* RNAi_2_ was inserted at the 40D locus. As insertions at the 40D locus can be associated with off-target effects, we did not pursue characterizing *dNT5B* RNAi_2_. We proceeded to determine knockdown efficacy of the remaining RNAi lines. Ubiquitous knockdown with *dNT5A* RNAi_1_ and RNAi_2_ reduced *dNT5A* mRNA level to 20 and 85% of the genetic background control in adulthood, respectively, as determined by using two independent primer pairs (Supplementary Fig. 2B, C). Knockdown of *dNT5A* with RNAi_1_ did not alter *dNT5B* and *dNT5C* levels; knockdown with RNAi_2_ slightly lowered also *dNT5C* level, although this finding did not withstand correction for multiple testing (Supplementary Fig. 2B, C). Ubiquitous knockdown using the remaining *dNT5B* RNAi line reduced *dNT5B* level to 40% of the genetic background control in adulthood (Supplementary Fig. 2D). This knockdown did not affect *dNT5A* or *dNT5C* levels (Supplementary Fig. 2D). Ubiquitous knockdown with *dNT5C* RNAi_1_ and RNAi_2_ reduced *dNT5C* levels to 35 and 25% of the genetic background control in adulthood, respectively (Supplementary Fig. 2E, F). Knockdown of *dNT5C* with RNAi_1_ did not alter *dNT5A* or *dNT5B* levels (Supplementary Fig. 2E). Knockdown with RNAi_2_ showed a slight reduction of *dNT5A*, but only detected with one primer pair (Supplementary Fig. 2F).

### Characterization of dNT5 models in neuropsychiatric disorders-related behaviors

**Fig. 2:**
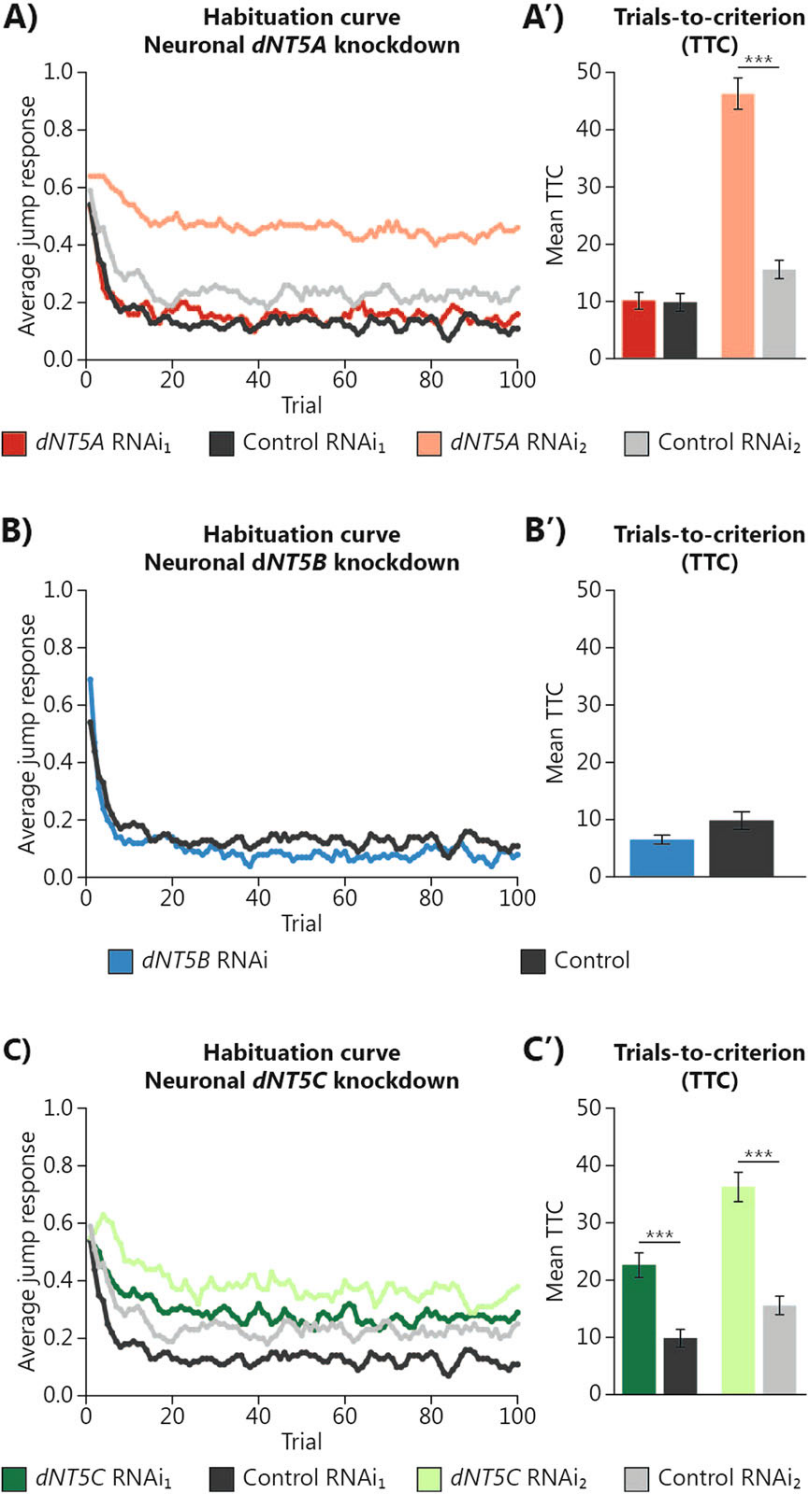
Neuronal manipulation of *dNT5A* and *dNT5C* expression causes habituation deficits. **A**–**C** Average jumping response of **A** *dNT5A*, **B** *dNT5B*, and **C** *dNT5C* pan-neuronal RNAi induction upon 100 consecutive light-off stimuli. A′, B′, C′ Quantification of trials-to-criterion (TTC). **A** Pan-neuronally induced *dNT5A* RNAi_2_ (*w*; *2xGMR*-*wIR*/*UAS*-*dNT5A RNAi*_*2*_*; nSyb*-*Gal4*, *UAS*-*Dcr2*, *n* = 176) habituated slower than the RNAi_2_ control (*w*; *2xGMR*-*wIR*/+*; nSyb*-*Gal4*, *UAS*-*Dcr2*, *n* = 123), while RNAi_1_ (*w*; *2xGMR*-*wIR; nSyb*-*Gal4*, *UAS*-*Dcr2*/*UAS*-*dNT5A RNAi*_*1*_, *n* = 122) habituated similarly to its control (*w*; *2xGMR*-*wIR; nSyb*-*Gal4*, *UAS*-*Dcr2*/+, *n* = 112). **B** Pan-neuronally induced *dNT5B* RNAi (*w*; *2xGMR*-*wIR; nSyb*-*Gal4*, *UAS*-*Dcr2*/*UAS*-*dNT5B RNAi*, *n* = 97) habituated similarly to control (*w*; *2xGMR*-*wIR; nSyb*-*Gal4*, *UAS*-*Dcr2*/+, *n* = 112). **C** Pan-neuronally induced *dNT5C* RNAi_1_ (*w*; *2xGMR*-*wIR; nSyb*-*Gal4*, *UAS*-*Dcr2*/*UAS*-*dNT5C RNAi*_*1*_, *n* = 161) and RNAi_2_ (*w*; *2xGMR*-*wIR*/*UAS*-*dNT5C RNAi*_*2*_*; nSyb*-*Gal4*, *UAS*-*Dcr2*, *n* = 138) habituated slower than their respective controls (RNAi_1_: *w*; *2xGMR*-*wIR; nSyb*-*Gal4*, *UAS*-*Dcr2*/+, *n* = 112; RNAi_2_: *w*; *2xGMR*-*wIR*/*+; nSyb*-*Gal4*, *UAS*-*Dcr2*, *n* = 123). All genotypes were tested together. Genetic background controls for *dNT5A*, *dNT5B*, and *dNT5C* RNAi_1_ as well as for *dNT5A* and *dNT5C* RNAi_2_ are identical, respectively, and re-plotted across panels for convenience. Error bars represent standard error of the mean. General linear model regression analysis with correction according the the number of RNAi tested was performed on the log-TTC values to obtain an adjusted *p*-value (*p*_adj_). (*p_adj_ < 0.05, **p_adj_ < 0.01, ***p_adj_ < 0.001). *N* = 3 biological replicates, minimum 30 flies/replicate.

Because of their association with neuropsychiatric disorders in humans, we further asked whether manipulating the expression of *Drosophila dNT5* genes in neurons would impact neuropsychiatric disorders-relevant behaviors. We first tested *dNT5* models in the light-off jump habituation paradigm, where the flies showed strong initial jumping reaction to non-threatening stimuli (light-off) which gradually weakened due to habituation learning^23,26^. Habituation is quantified as TTC, the number of stimuli needed to reach habituation criterion (see “Methods” section). Pan-neuronal *dNT5A* knockdown with RNAi_1_ showed a TTC similar to the one observed in the genetic background control (*p*_adj_ > 0.05) (Fig. 2A and Supplementary Table 3), while use of RNAi_2_, caused severe habituation deficits with a 3-fold increased TTC value compared to its control (*p*_adj_ < 0.001) (Fig. 2A and Supplementary Table 3). Pan-neuronal *dNT5B* knockdown did not affect habituation (*p*_adj_ > 0.05) (Fig. 2B and Supplementary Table 3). Pan-neuronal *dNT5C* knockdown caused habituation deficits with both RNAi lines (Fig. 2C), with both knockdown models showing more than a 2-fold increase of the TTC value (RNAi_1_ *p*_adj_ < 0.001; RNAi_2_ *p*_adj_ < 0.001) (Fig. 2C’ and Supplementary Table 3).

**Fig. 3:**
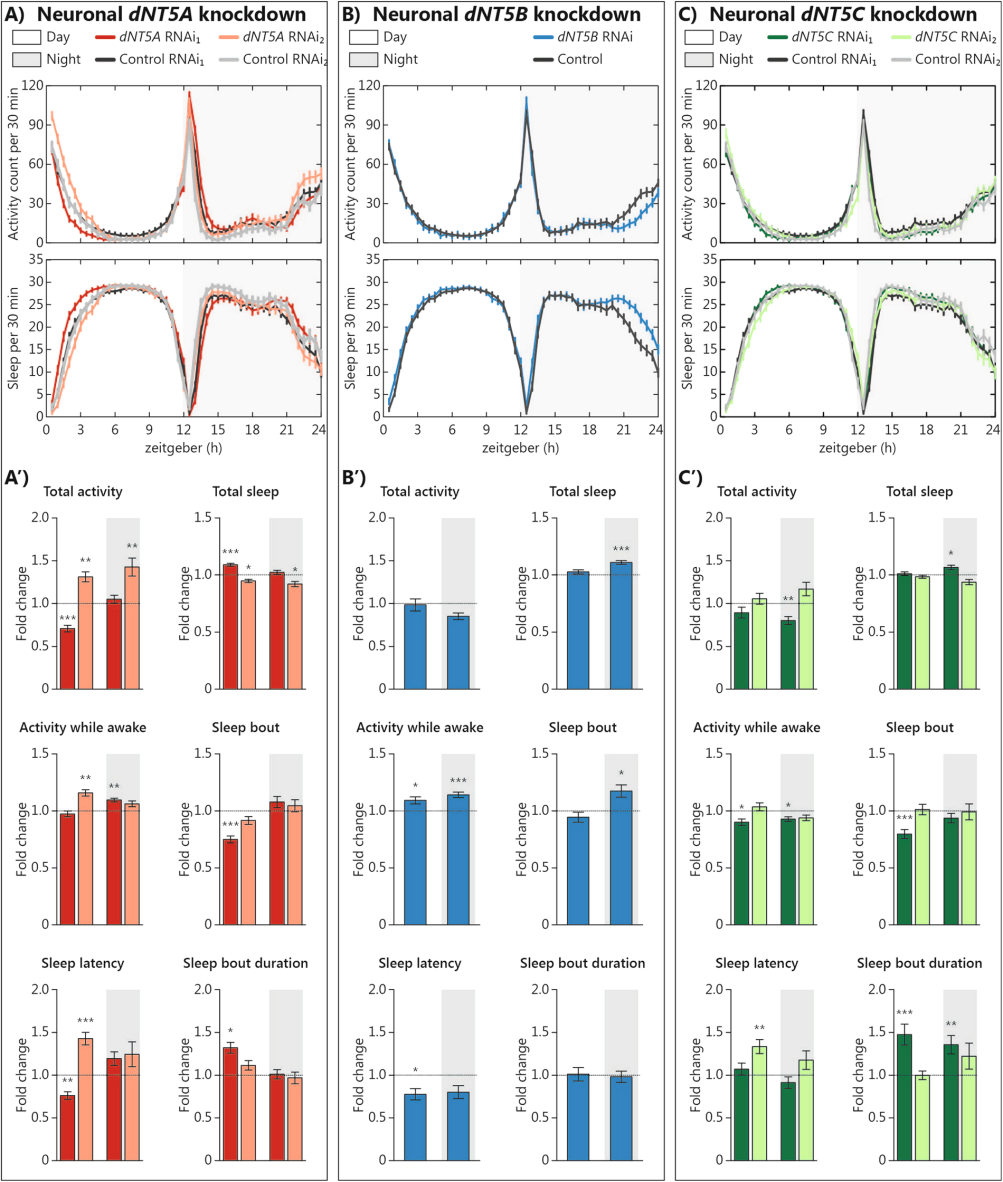
Neuronal manipulation *dNT5A*, *dNT5B*, and *dNT5C* only slightly affected activity and sleep. **A**–**C** Activity and sleep plot of pan-neural manipulation of **A** *dNT5A* (RNAi_1_: *w*, *UAS*-*Dcr2*;; *nSyb*-*Gal4*/*UAS*-*dNT5A RNAi*_*1*_, *n* = 84; RNAi_2_: *w*, *UAS*-*Dcr2*; *UAS*-*dNT5A RNAi*_*2*_; *nSyb*-*Gal4*, *n* = 77), **B** *dNT5B* (*w*, *UAS*-*Dcr2*;; *nSyb*-*Gal4*/*UAS*-*dNT5B RNAi*, *n* = 71), and **C** *dNT5C* (RNAi_1_: *w*, *UAS*-*Dcr2*;; *nSyb*-*Gal4*/*UAS*-*dNT5C RNAi*_*1*_, *n* = 86; RNAi_2_: *w*, *UAS*-*Dcr2*; *UAS*-*dNT5C RNAi*_*2*_; *nSyb*-*Gal4*, *n* = 61). All genotypes were tested together. Genetic background controls for *dNT5A*, *dNT5B*, and *dNT5C* RNAi_1_ as well as for *dNT5A* and *dNT5C* RNAi_2_ are identical, respectively, and re-plotted across panels for convenience. A′, B′, C′ Quantification of activity and sleep parameters, normalized to respective control (RNAi_1_: *w*, *UAS*-*Dcr2*;; *nSyb*-*Gal4*/+, *n* = 88; RNAi_2_: *w*, *UAS*-*Dcr2*; +; *nSyb*-*Gal4*, *n* = 62). Error bars represent standard error of the mean. One-way analysis of variance (ANOVA) followed by Dunnett’s post-hoc test was used to obtain an adjusted *p*-value (*p*_adj_) (*p_adj_ < 0.05, **p_adj_ < 0.01, ***p_adj_ < 0.001). *N* = 3 biological replicates, minimum 20 flies/replicate.

Increased locomotor activity and reduced sleep have been previously shown to characterize *Drosophila* models of ADHD^21,22^. Furthermore, increased sleep in male flies has been reported in a *Drosophila* model of schizophrenia^36^. When monitoring activity and sleep of *dNT5* genes knockdown, we found slight activity and sleep differences compared to controls (Fig. 3A–C). Pan-neuronal *dNT5A* knockdown with RNAi_1_ caused 30% less activity counts (*p*_adj_ < 0.001) and 10% increased sleep (*p*_adj_ < 0.001) during the day (Fig. 3A′ and Supplementary Table 4A). Conversely, knockdown with RNAi_2_ increased activity counts by 30% (*p*_adj_ < 0.01) and reduced sleep by 5% (*p*_adj_ < 0.05) during the day; during the night, the RNAi_2_ construct increased activity counts by 40% (*p*_adj_ < 0.01) and reduced sleep by 10% (*p*_adj_ < 0.05) (Fig. 3A′ and Supplementary Table 4B). Pan-neuronal *dNT5B* knockdown showed 10% increased total night sleep (*p*_adj_ < 0.001) (Fig. 3B′ and Supplementary Table 4A). Pan-neuronal *dNT5C* knockdown with RNAi_1_ showed 20% reduced total night activity (*p*_adj_ < 0.01) and 5% increased night sleep (*p*_adj_ < 0.05) (Fig. 3C′ and Supplementary Table 4A), but knockdown with RNAi_2_ showed similar total activity and sleep to the control (Fig. 3C′ and Supplementary Table 4B). Altogether, manipulation of the different dNT5s did affect activity and sleep, with knockdown consistently leading to reduced activity and/or increased sleep, but always in a relatively mild fashion.

### Characterization of synapse morphology in dNT5 models

**Fig. 4:**
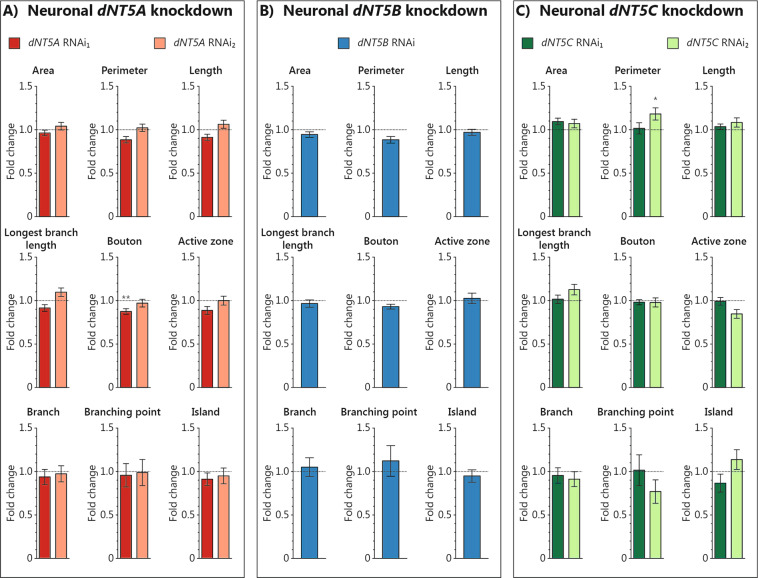
Neuronal manipulation of *dNT5A*, *dNT5B*, and *dNT5C* did not affect development of synaptic terminals at the *Drosophila* neuromuscular junction (NMJ). **A**–**C** Synaptic NMJ parameters of pan-neuronal manipulation of **A** *dNT5A* (RNAi_1_: *w*, *UAS*-*Dcr2*;; *nSyb*-*Gal4*/*UAS*-*dNT5A RNAi*_*1*_, *n* = 34; RNAi_2_: *w*, *UAS*-*Dcr2*; *UAS*-*dNT5A RNAi*_*2*_; *nSyb*-*Gal4*, *n* = 33), **B** *dNT5B* (*w*, *UAS*-*Dcr2*;; *nSyb*-*Gal4*/*UAS*-*dNT5B RNAi*, *n* = 29), and **C** *dNT5C* (RNAi_1_: *w*, *UAS*-*Dcr2*;; *nSyb*-*Gal4*/*UAS*-*dNT5C RNAi*_*1*_, *n* = 15; RNAi_2_: *w*, *UAS*-*Dcr2*; *UAS*-*dNT5C RNAi*_*2*_; *nSyb*-*Gal4*, *n* = 26). All values were normalized to respective control (*dNT5A*, *dNT5B*, and *dNT5C* RNAi_1_: *w*, *UAS*-*Dcr2*;; *nSyb*-*Gal4*/+, *n* = 36; *dNT5A* and *dNT5C* RNAi_2_: *w*, *UAS*-*Dcr2*; +; *nSyb*-*Gal4*, *n* = 40). Representative NMJ terminal microscope images are presented in Supplementary Fig. 3. Error bars represent standard error of the mean. One-way analysis of variance (ANOVA) followed by Dunnett’s post-hoc test was used to obtain an adjusted *p*-value (*p*_adj_). (*p_adj_ < 0.05, **p_adj_ < 0.01, ***p_adj_ < 0.001). *N* = 5–10 animals/genotype.

In addition to the behavioral assays, we also examined synaptic morphology in the dNT5 neuronal knockdown models. Disruption of synaptic development and function has been implicated in multiple psychiatric disorders^37–39^. For example, the schizophrenia risk gene *DISC1* has been shown to regulate synaptogenesis at the *Drosophila* neuromuscular junction (NMJ)^40^, a popular model synapse in the fruit fly^41,42^. In a comprehensive assessment of eight interdependent morphological parameters and the number of active zones, synaptic terminals in the dNT5 models developed largely comparable to their controls (Fig. 4A–C and Supplementary Fig. 3). Pan-neuronal *dNT5A* knockdown with RNAi_1_ showed 12% less synaptic boutons than control animals (*p*_adj_ < 0.01) (Fig. 4A and Supplementary Table 5A, B), while pan-neuronal *dNT5C* knockdown with RNAi_2_ showed 18% larger perimeter than its control (*p*_adj_ < 0.05) (Fig. 2C and Supplementary Table 5A, B). The dNT5 proteins thus appear to only have very minor, if any, effect on NMJ development and morphology.

## Discussion

Despite multiple associations to neuropsychiatric disorders, the role of the cytosolic 5′-nucleotidase II (cNT5-II) family of enzymes in nervous system functioning is still largely unknown. Prior to the current study, among the cNT5-II family members, a neuronal function had only been demonstrated for *NT5C2*; the neuronal function of *NT5DC1*, *NT5DC2*, *NT5DC3*, and *NT5DC4* was yet to be investigated. Here, we investigated the neuronal expression and function of *NT5C2*, *NT5DC1*, *NT5DC2*, *NT5DC3*, and *NT5DC4* orthologous genes in *Drosophila*. Our data showed that the cNT5-II (*dNT5*) genes are expressed in the brain and that altering their neuronal expression in *Drosophila* is linked to altered habituation, activity, and/or sleep. Our findings further establish the role of the cNT5-II family, particularly *NT5DC2* and/or *NT5DC3*, in the nervous system.

Among the *dNT5* genes, *dNT5C*, the orthologue of *NT5DC2* and *NT5DC3*, showed the strongest evidence for an important neuronal role. Its expression was enriched in the adult brain. This finding is in line with publicly available single cell RNA sequencing data, which showed more *dNT5C*-positive cells than *dNT5A*-positive and *dNT5B*-positive ones in the brain^43^. Consistent with the robust expression in the brain, *dNT5C* also caused the strongest deficits in habituation learning upon pan-neuronal knockdown. Considering that habituation correlates with cognitive performance^44^ and *NT5DC2* is linked to cognitive performance and educational attainment through multiple GWASs^9^, our findings also reinforce *NT5DC2*’s association with cognitive performance. *NT5DC2* knockdown has been shown to increase the synthesis of catecholamines in a cellular model^12^. Increased monoaminergic signaling in the brain has consistently been shown to promote wakefulness across species^45,46^. Indeed, we found mild effects of *dNT5C* pan-neuronal knockdown on activity and sleep, though predominantly for the RNAi line producing the stronger knockdown. More generally, the habituation test appeared to be the most sensitive to *dNT5C* pan-neuronal knockdown. *dNT5C* knockdown has less influence on locomotor activity, sleep, and morphology of NMJ terminals. Further research is warranted to follow-up on the behavioral effects of *NT5DC2* and the role of monoaminergic signaling therein.

Out of all cNT5-II family members, only *NT5C2* had been studied for its neuronal role prior to our study. *NT5C2* was shown to regulate protein translation in neural progenitor cells, and neuronal expression of *dNT5B*, orthologue of *NT5C2* and *NT5DC4*, was shown to cause motor defects in a climbing assay^2^. Moreover, *NT5C2* has the strongest association to neuropsychiatric disorders among the cNT5-II family members (Supplementary Table 1). Despite such evidence, neuronal knockdown of *dNT5B* did not alter neuropsychiatric disorder-related behaviors in the current study. It should be noted, however, that the outcome was based on the data from only one RNAi construct. Furthermore, as this induced *dNT5B* RNAi reduced *dNT5B* mRNA level by half, the knockdown might have been not efficient enough to alter behavior and synaptic morphology. In addition, despite previous report of motor defects upon neuronal *dNT5B* knockdown, we did not observe significantly affected motor function in our habituation assays, as illustrated by the effective initial jump response of *dNT5B* knockdown flies and similar number of flies jumping throughout the habituation assay. The difference between the two studies may be explained by the different behaviors (climbing versus startle response) that exploit different neuronal circuits, and hence are not directly comparable. Also, the two studies employed different genetic tools: Duarte et al.^2^ used *elav*-*Gal4* in their study, while we used another pan-neuronal driver, *nSyb*-*Gal4*. While both drivers are widely used pan-neuronal drivers, differences in onset, promotor strength, and/or persistence in adulthood are likely to exist and can lead to different levels of knockdown in neuronal subpopulations.

For *dNT5A*, the orthologue of *NT5DC1*, we found that knockdown with RNAi_2_, associated with weaker knockdown, was accompanied by severe habituation deficits, while knockdown with RNAi_1_, associated with stronger knockdown, showed normal habituation. This is surprising, but following explanations might be given. First, RNAi-mediated silencing can also (partly) occur through repression of translation^47^. The degree of knockdown upon induction of RNAi_2_ might potentially be an underestimation. Second, despite the high predicted specificity of all RNAi lines utilized in this study, we cannot formally exclude an off-target effect. Third, the knockdown observed using the ubiquitous driver might incompletely reflect the knockdown induced by the neuronal driver. Fourth, knockdown with RNAi_2_ was accompanied by nominally reduced expression of *dNT5C*. Since *dNT5C* knockdown was associated with strong habituation deficits, reduced *dNT5C* level may potentially contribute to the habituation deficits detected in pan-neuronal RNAi_2_-mediated *dNT5A* knockdown. Future studies should investigate whether habituation deficits in this model was caused by altered *dNT5C* level or purely caused by altered *dNT5A* level. Prior to our study, *NT5DC1* had only been linked to cognitive function and neuropsychiatric disorders through GWASs^9^. Our findings on *dNT5A* function in habituation learning, activity, and sleep support the hypothesis of *NT5DC1* having a role in neuropsychiatric disorders and cognitive function.

Our findings should be viewed in the context of the strengths and limitations of this study. We used the animal model *Drosophila melanogaster* combined with a reverse genetics approach to study the function of *dNT5* genes in behavior and synapse morphology to complement genetics studies and non-invasive research in humans. Using qRT-PCR, we determined the enrichment of each dNT5 genes in the brain relative to the rest of the body. Since qRT-PCR only allows to compare expression of the same gene or amplicon in multiple conditions, it is not possible to compare expression of the three genes with each other. An objective method that allow comparison is RNAseq. Such large scale genomic data is publicly available^43^, although it is not available at the time resolution of our experiments. The fly model provides a tissue-specific gene manipulation system combined with RNAi-mediated knockdown, which allowed the study of *dNT5* gene functions specifically in neurons. However, RNAi-mediated knockdown is predominantly dependent on the specificity and efficiency of the RNAi construct. While we carefully chose RNAi lines with high s19 score (>0.99) to ensure specificity and incorporated *UAS*-*Dcr2* element to enhance knockdown efficiency^25^, knockdown induced might not be sufficient to cause stronger effects, especially on activity and sleep. Moreover, in the current study, we only set out to study knockdown of the *dNT5* genes, extrapolating from the few findings in humans linked to malfunctioning of the gene/protein and reduced gene expression. However, most links to human behavior come from GWAS, where it is still difficult to define the direction of effect of associated genetic variants. We therefore cannot exclude a role for overexpression of the dNT5 genes in the phenotypes studied here. More research is warranted to investigate the consequence of dNT5 gene overexpression in psychiatric disorders-related behavior.

In summary, we here confirmed existing evidence for a neuronal role of cNT5-II family members, and extended knowledge by reporting such a role also for *NT5DC1*, *NT5DC2*, and *NT5DC3* using *Drosophila* as a model. The *dNT5* genes impact habituation learning, activity, and sleep, providing supporting evidence that cNT5-II family genes can contribute to the etiology of neuropsychiatric disorders. Although research so far might have only been focused on the neurobiology of *NT5C2*, studying the neuronal role of other cNT5-II family members, especially *NT5DC2* and *NT5DC3*, can provide additional insight into the biology underlying the neuropsychiatric disorders.

## Supplementary information

Supplementary Materials

Supplementary Figure 1

Supplementary Figure 2

Supplementary Figure 3
