# Supplementary material for: Investigating cytosolic 5′-nucleotidase II family genes as candidates for neuropsychiatric disorders in Drosophila (114/150 chr)
Source: Transl Psychiatry. 2021 Jan 18;11:55. doi: 10.1038/s41398-020-01149-x (PMC7813868; doi:10.1038/s41398-020-01149-x)
Supplement: Supplementary file 1 — Supplementary Materials [file 41398_2020_1149_MOESM1_ESM.docx]

**Supplementary materials for:**

**Investigating cytosolic 5’-nucleotidase II family genes as candidates for neuropsychiatric disorders in *Drosophila***

Euginia L Singgih, BSc^1^, Monique van der Voet, PhD^1^, Marlies Schimmel-Naber, BSc^1^, Emma L Brinkmann, BSc^1^, Annette Schenck, PhD^1#^, Barbara Franke, PhD^1,2#^

^1^Radboud university medical center, Donders Institute for Brain, Cognition and Behaviour, Department of Human Genetics, Nijmegen, The Netherlands

^2^Radboud university medical center, Donders Institute for Brain, Cognition and Behaviour, Department of Psychiatry, Nijmegen, The Netherlands

^#^shared final responsibility

Corresponding author: Barbara Franke ([barbara.franke@radboudumc.nl](mailto:barbara.franke@radboudumc.nl))

This document contains:

- Supplementary method
- Supplementary Figure 1-3
- Supplementary Table 1-5

**Supplementary method**

***Validation of RNAi lines***

*DNA isolation and diagnostic PCR*

As the UAS RNAi constructs from VDRC KK collection may have inserted at 30B and/or 40D sites, whereby the insertion at 40D might drive ectopic *tio* expression and associated off-target effects [1, 2], we performed diagnostic PCR to map the insertion sites of each of the constructs used in this study. A single adult male fly was collected from each genotype, snap-frozen in liquid nitrogen, and crushed in 50ul crushing buffer (10 mM Tris-HCl pH 8.2, 1mM EDTA, 25mM NaCl) containing Proteinase K (0.1ug/0.3U) (Thermo Fisher Scientific, Lithuania). The mixture was then incubated for 30 minutes at 37°C, continued with 2 minutes at 95°C to inactivate Proteinase K. The supernatant was collected and subsequently used for a PCR-based diagnostic assay as described [1] using AmpliTaq360 (Thermo Fisher Scientific, Lithuania). Primer sequences are available in **Supplementary Table 2**. A touchdown PCR program was used (94°C 20s denaturation, 68°C 45s annealing, 72°C 60s elongation, annealing temperature was lowered for 0.5°C every cycle for 8 cycle; continued with 94°C 20s denaturation, 62°C 30s annealing, 72°C 60s elongation for 30 cycle). The PCR was performed with a Dyad Thermal Cycler (BioRad). The PCR product was then analyzed by electrophoresis using non-denaturing 1.5% agarose gel in 0.5x TBE buffer containing GelRed (Biotium, Fremont, CA). The image of the gel was captured with an Imager (G-Box, Syngene, UK).

*RNA isolation and qRT-PCR*

The adult male progenies at age 2-3 days past eclosion were collected and snap-frozen in liquid nitrogen. The total RNA was extracted with RNeasy Lipid Tissue Mini kit (Qiagen, Germany). Complementary DNA (cDNA) was synthesized from total RNA with the iScript kit (BioRad, CA, USA). The cDNA was diluted 5x in purified water and subjected to qRT-PCR using Power SYBR® Green PCR master mix (Thermo Fisher Scientific, UK) with QuantStudio 5 (Applied Biosystems) in 384-well plate format (primer sequences available in **Supplementary Table 2**). Primer sets were designed with Primer3 [3], unless specified. Expression of *eIF-1A* and *αTub84B* were used as internal control. Cycle threshold (C_T_) values were determined with QuantStudio Design and Analysis Software 1.4.3 (Applied Biosystems), and the difference in expression level was determined using the ΔΔC_T_ method [4]. Statistical analysis was performed with Graphpad (San Diego, CA).

**Supplementary Figures**

**
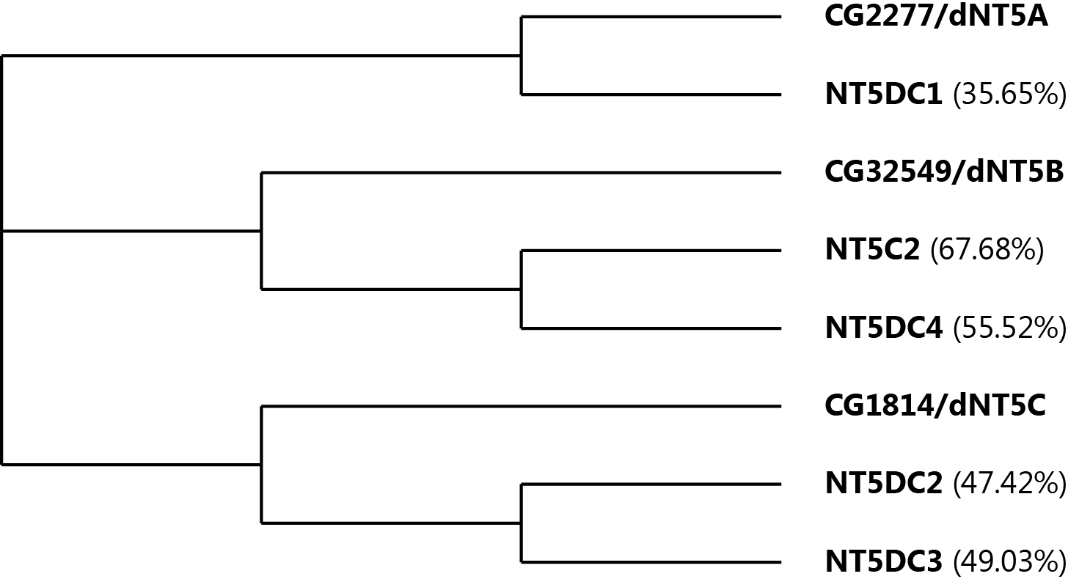
**

**Supplementary Figure 1.** *Drosophila* orthologues of cNT5-II genes. *CG2277*/*dNT5A* is the orthologue of *NT5DC1*; *CG32549*/*dNT5B* of *NT5C2* and *NT5DC4*; *CG1814*/*dNT5C* of *NT5DC2* and *NT5DC3*. The percentage amino acid identity of the human cNT5-II genes to their *Drosophila* orthologue is indicated in brackets behind each human gene. Phylogenetic tree and percentage amino acid identity were generated with Clustal Omega [5].


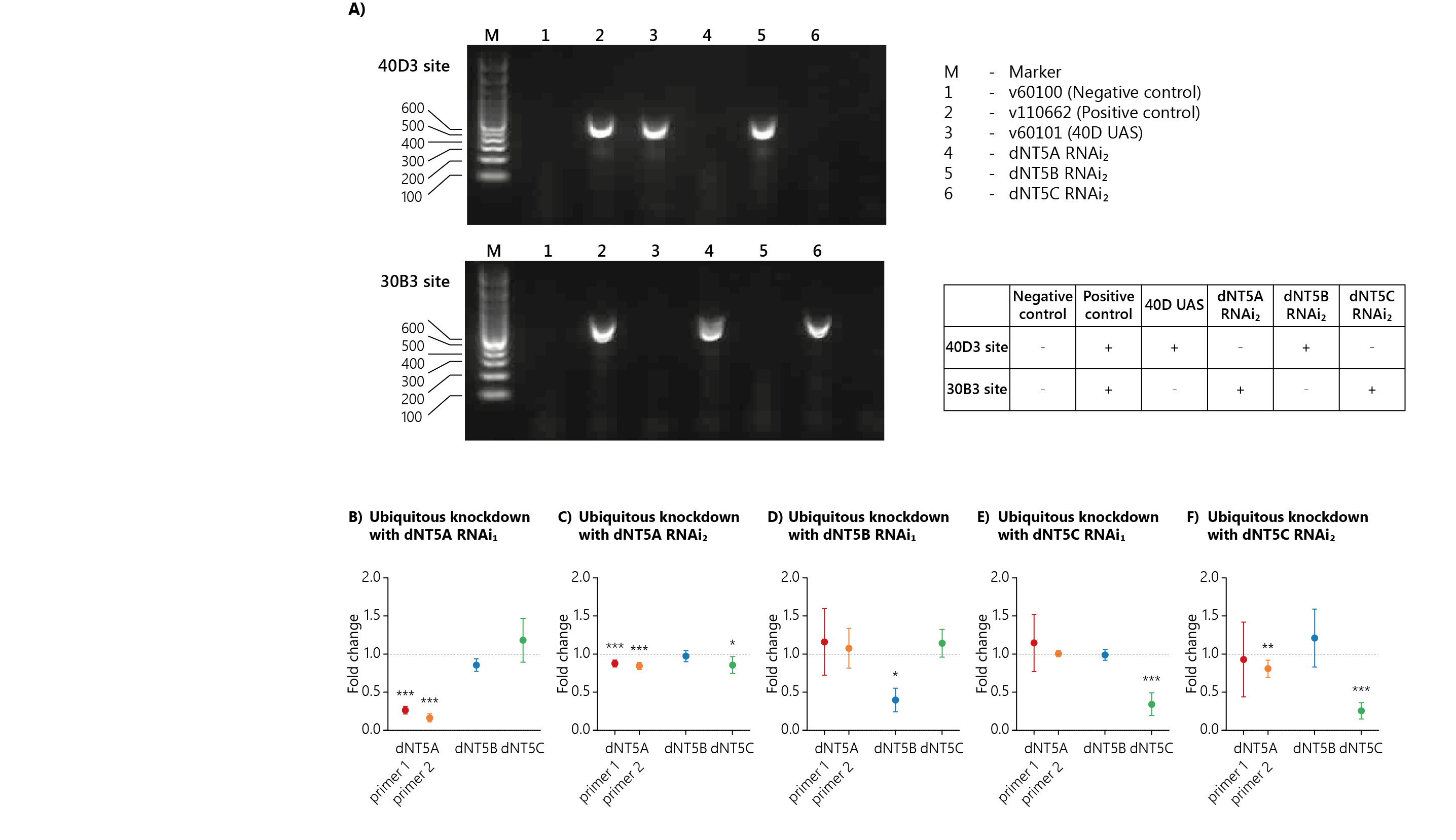


**Supplementary Figure 2.** Characterization of *UAS*-RNAi lines used in this study. (A) Agarose gel electrophoresis showing the result of diagnostic PCR to determine the insertion of *UAS* constructs at 40D and 30B sites. *UAS*-*dNT5A* *RNAi_2_* and *UAS*-*dNT5C* *RNAi_2_* constructs were inserted at 30B position while *UAS*-*dNT5B* *RNAi_2_* at 40D. Consequently, the *UAS*-*dNT5B* *RNAi_2_* was not used further due to expected off-target effect. (B-F) Relative expression of *dNT5* genes upon ubiquitous knockdown with (B) *dNT5A* RNAi_1_ (*w*;; *da.G32*-*Gal4*/*UAS*-*dNT5A* *RNAi_1_*), (C) *dNT5B* RNAi_2_ (*w*; *UAS*-*dNT5A* *RNAi_2_*; *da.G32*-*Gal4*), (D) *dNT5B* RNAi_1_ (*w*;; *da.G32*-*Gal4*/*UAS*-*dNT5B* *RNAi_1_*), (E) *dNT5C* RNAi_1_ (*w*;; *da.G32*-*Gal4*/*UAS*-*dNT5C* *RNAi_1_*), and (F) *dNT5C* RNAi_2_ (w; *UAS*-*dNT5C* *RNAi_2_*; *da.G32*-*Gal4*), as measured by qRT-PCR. Two primer sets were used to measure the level of *dNT5A* upon knockdown. All values were normalized to the respective control (*w*;; *da.G32*-*Gal4*/+). (B) Ubiquitous knockdown of *dNT5A* with RNAi_1_ reduced *dNT5A* expression level, while *dNT5B* and *dNT5C* levels were unaffected. (C) Ubiquitous knockdown of *dNT5A* with RNAi_2_ reduced *dNT5A* expression level; *dNT5B* level was not affected, but *dNT5C* level appeared to be slightly reduced. (D) Ubiquitous knockdown of *dNT5B* with RNAi_1_ reduced *dNT5B* expression level, while *dNT5A* and *dNT5C* levels were unaffected. (E) Ubiquitous knockdown of *dNT5C* with RNAi_1_ reduced *dNT5C* expression level, while *dNT5A* and *dNT5C* levels were not affected. (F) Ubiquitous knockdown of *dNT5C* with RNAi_2_ reduced *dNT5C* expression level; *dNT5A* level appeared to be slightly reduced but only with one primer set, while *dNT5B* level was not affected. For *dNT5A* RNAi_1_ and *dNT5A* RNAi_2_ n=6 biological replicates were performed with n=3 technical replicates each. For *dNT5B* RNAi_1_, *dNT5C* RNAi_1_, and *dNT5C* RNAi_2_, n=3 biological replicates were performed with n=3 technical replicates each. Error bars represent 95% confidence intervals. (*p<0.05, **p<0.01, ***p<0.001).


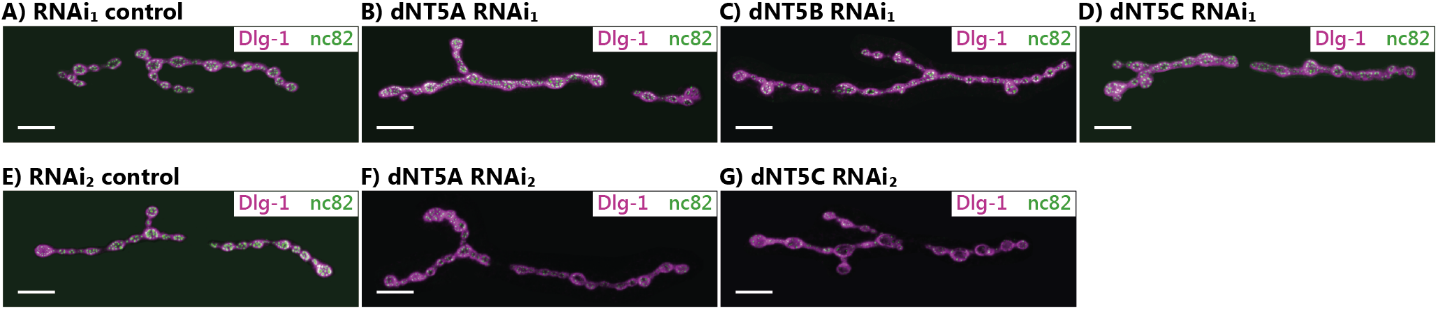


**Supplementary Figure 3.** Neuronal manipulation of *dNT5A*, *dNT5B*, and *dNT5C* did not alter morphology of neuromuscular junction (NMJ) synaptic terminals. Representative microscope images of NMJ terminal, co-immunolabeled with anti-Dlg-1 and anti-nc82. (A) Control for *dNT5A*, *dNT5B*, and *dNT5C* RNAi_1_ (*w*, *UAS*-*Dcr2*; ; *nSyb*-*Gal4*/+). (B) *dNT5A* RNAi_1_ (*w*, *UAS*-*Dcr2*; ; *nSyb*-*Gal4*/*UAS*-*dNT5A* *RNAi_1­_*). (C) *dNT5B* RNAi (*w*, *UAS*-*Dcr2*; ; *nSyb*-*Gal4*/*UAS*-*dNT5B RNAi*). (D) *dNT5C* RNAi_1_ (*w*, *UAS*-*Dcr2*; ; *nSyb*-*Gal4*/*UAS*-*dNT5C* *RNAi_1_*). (E) Control for *dNT5A* and *dNT5C* RNAi_2_ (*w*, *UAS*-*Dcr2*; +; *nSyb*-*Gal4*). (F) *dNT5A* RNAi_2_ (*w*, *UAS*-*Dcr2*; *UAS*-*dNT5A* *RNAi_2_*; *nSyb*-*Gal4*). (G) *dNT5C* RNAi_2_ (*w*, *UAS*-*Dcr2*; *UAS*-*dNT5C* *RNAi_2_*; *nSyb*-*Gal4*). Scale bars represent 10 µm.

**Supplementary Tables**

**Supplementary Table 1A.** GWAS atlas output for *NT5C2* for all psychiatric GWASs (n=321), retrieved on February 17^th^, 2020.

| **atlas ID** | **PMID** | **Year** | **Domain** | **Trait** | **P-value** | **N** |
| --- | --- | --- | --- | --- | --- | --- |
| 4070 | BioRxiv: https://doi.org/10.1101/261081 | 2018 | Psychiatric | Ever smoker | 6.66E-20 | 518633 |
| 3654 | 31427789 | 2019 | Psychiatric | Smoking status: Never | 9.94E-19 | 384964 |
| 3236 | 31427789 | 2019 | Psychiatric | Past tobacco smoking | 1.11E-14 | 355594 |
| 4314 | 30643251 | 2019 | Psychiatric | Ever smoked regulary | 5.42E-14 | 262990 |
| 3982 | 29483656 | 2018 | Psychiatric | Schizophrenia | 5.72E-14 | 105318 |
| 11 | 25056061 | 2014 | Psychiatric | Schizophrenia | 5.96E-14 | 82315 |
| 13 | 24280982 | 2014 | Psychiatric | Schizophrenia vs Bipolar disorder | 5.96E-14 | 16381 |
| 3425 | 31427789 | 2019 | Psychiatric | Ever smoked | 1.64E-13 | 385013 |
| 3996 | 29500382 | 2018 | Psychiatric | Nervous feelings (NERV-FEEL) | 2.10E-13 | 264858 |
| 3798 | 29942085 | 2018 | Psychiatric | Worry subcluster | 2.55E-13 | 348219 |
| 3289 | 31427789 | 2019 | Psychiatric | Nervous feelings | 3.95E-13 | 376368 |
| 3997 | 29500382 | 2018 | Psychiatric | Worrier / anxious feelings (WORRY) | 4.63E-13 | 264646 |
| 4038 | 29906448 | 2018 | Psychiatric | Schizophrenia | 1.18E-12 | 87491 |
| 3290 | 31427789 | 2019 | Psychiatric | Worrier / anxious feelings | 1.50E-12 | 376411 |
| 4040 | 29906448 | 2018 | Psychiatric | Schizophrenia/Bipolar disorder | 3.39E-12 | 107620 |
| 10 | 23974872 | 2013 | Psychiatric | Schizophrenia | 1.82E-11 | 32143 |
| 3439 | 31427789 | 2019 | Psychiatric | Tobacco smoking | 1.12E-09 | 97172 |
| 4270 | 30867560 | 2019 | Psychiatric | Anxiety/tension factors | 3.70E-09 | 270059 |
| 4271 | 30867560 | 2019 | Psychiatric | Worry/vulnerability factors | 3.70E-09 | 270059 |
| 3235 | 31427789 | 2019 | Psychiatric | Current tobacco smoking | 2.44E-08 | 386150 |
| 3744 | 31427789 | 2019 | Psychiatric | Cannabis use - Ever taken cannabis | 3.20E-08 | 126632 |
| 9 | 21926974 | 2011 | Psychiatric | Schizophrenia | 2.95E-07 | 21856 |
| 12 | 24280982 | 2014 | Psychiatric | Schizophrenia/Bipolar disorder | 5.44E-07 | 39202 |
| 1191 | 23453885 | 2013 | Psychiatric | PGC cross disorder | 6.03E-07 | 61220 |
| 3296 | 31427789 | 2019 | Psychiatric | Risk taking | 9.95E-07 | 372651 |
| 3795 | 29942085 | 2018 | Psychiatric | Neuroticism | 1.54E-06 | 390278 |
| 3729 | 31427789 | 2019 | Psychiatric | Depression - Age at first episode of depression | 1.77E-06 | 65776 |
| 3990 | 29500382 | 2018 | Psychiatric | Neuroticism sum score | 2.49E-06 | 380506 |
| 3265 | 31427789 | 2019 | Psychiatric | Average weekly spirits intake | 5.08E-06 | 273678 |
| 3335 | 31427789 | 2019 | Psychiatric | Light smokers, at least 100 smokes in lifetime | 1.04E-05 | 105610 |
| 3417 | 31427789 | 2019 | Psychiatric | Neuroticism score | 1.15E-05 | 312740 |
| 3791 | 30804565 | 2019 | Psychiatric | Ease of getting up in the morning | 1.70E-05 | 385949 |
| 4368 | 30150663 | 2018 | Psychiatric | Cannabis use | 2.04E-05 | 162082 |
| 4249 | 30952852 | 2019 | Psychiatric | Diurnal inactivity duration | 2.19E-05 | 84757 |
| 3229 | 31427789 | 2019 | Psychiatric | Getting up in morning | 2.63E-05 | 385494 |
| 4041 | 29906448 | 2018 | Psychiatric | Schizophrenia vs Bipolar disorder | 3.59E-05 | 38855 |
| 3998 | 29500382 | 2018 | Psychiatric | Tense / 'highly strung' (TENSE) | 4.35E-05 | 263635 |
| 3291 | 31427789 | 2019 | Psychiatric | Tense / 'highly strung' | 8.87E-05 | 374129 |
| 4000 | 29500382 | 2018 | Psychiatric | Suffer from 'nerves' (SUF-NERV) | 1.35E-04 | 262321 |
| **Bonferroni correction threshold** | | | | | **1.56E-04** |  |
| 3293 | 31427789 | 2019 | Psychiatric | Suffer from 'nerves' | 1.70E-04 | 372273 |
| 4293 | 30718901 | 2019 | Psychiatric | Depression | 1.93E-04 | 500199 |
| 3352 | 31427789 | 2019 | Psychiatric | Age started smoking in former smokers | 2.59E-04 | 94891 |
| 4087 | 29255261 | 2018 | Psychiatric | Neuroticism | 3.72E-04 | 329821 |
| 3357 | 31427789 | 2019 | Psychiatric | Likelihood of resuming smoking | 4.32E-04 | 93128 |
| 4321 | 30643256 | 2019 | Psychiatric | Neuroticism (univariate) | 5.11E-04 | 523783 |
| 3292 | 31427789 | 2019 | Psychiatric | Worry too long after embarrassment | 5.39E-04 | 370660 |
| 4337 | 30531941 | 2018 | Psychiatric | Sleep durataion | 5.62E-04 | 91105 |
| 3286 | 31427789 | 2019 | Psychiatric | Irritability | 6.83E-04 | 369232 |
| 3789 | 30804565 | 2019 | Psychiatric | Morningness | 7.12E-04 | 345552 |
| 4338 | 30531941 | 2018 | Psychiatric | Sleep durataion (conditioning sex and BMI) | 8.00E-04 | 91105 |
| 3999 | 29500382 | 2018 | Psychiatric | Worry too long after embarrassment (WORR-EMB) | 8.75E-04 | 261094 |
| 3299 | 31427789 | 2019 | Psychiatric | Frequency of tenseness / restlessness in last 2 weeks | 0.00105 | 371869 |
| 3230 | 31427789 | 2019 | Psychiatric | Morning/evening person (chronotype) | 0.00142 | 345148 |
| 3268 | 31427789 | 2019 | Psychiatric | Alcohol intake versus 10 years previously | 0.00148 | 357907 |
| 3756 | 31427789 | 2019 | Psychiatric | Mental distress - Ever suffered mental distress preventing usual activities | 0.00149 | 125147 |
| 3239 | 31427789 | 2019 | Psychiatric | Exposure to tobacco smoke outside home | 0.00194 | 327794 |
| 4253 | 30952852 | 2019 | Psychiatric | Sleep duration (mean) | 0.00195 | 85449 |
| 19 | 20418890 | 2010 | Psychiatric | Ever versus never beeing a regular smoker | 0.00211 | 74035 |
| 3994 | 29500382 | 2018 | Psychiatric | Sensitivity / hurt feelings (HURT) | 0.00223 | 264144 |
| 4294 | 30696823 | 2019 | Psychiatric | Chronotype | 0.00259 | 449732 |
| 4011 | 29662059 | 2018 | Psychiatric | Broad depression | 0.00409 | 322580 |
| 3793 | 30804565 | 2019 | Psychiatric | Sleep duration | 0.00470 | 384317 |
| 3728 | 31427789 | 2019 | Psychiatric | Anxiety - Ever worried more than most people would in similar situation | 0.00576 | 106215 |
| 3287 | 31427789 | 2019 | Psychiatric | Sensitivity / hurt feelings | 0.00627 | 375272 |
| 3774 | 31427789 | 2019 | Psychiatric | Traumatic events - Been in aconfiding relationship as an adult | 0.00713 | 123584 |
| 3993 | 29500382 | 2018 | Psychiatric | Irritability (IRR) | 0.00736 | 260369 |
| 3233 | 31427789 | 2019 | Psychiatric | Snoring | 0.00758 | 359498 |
| 3267 | 31427789 | 2019 | Psychiatric | Alcohol usually taken with meals | 0.00819 | 197613 |
| 4269 | 30867560 | 2019 | Psychiatric | Neuroticism general factor | 0.00831 | 270059 |
| 4295 | 30696823 | 2019 | Psychiatric | Morning person (binary) | 0.00849 | 403195 |
| 3655 | 31427789 | 2019 | Psychiatric | Smoking status: Previous vs Current | 0.00874 | 177025 |
| 4014 | 29700475 | 2018 | Psychiatric | Major depressive disorder | 0.01024 | 173005 |
| 4256 | 30952852 | 2019 | Psychiatric | Sleep midpoint | 0.01031 | 84810 |
| 3726 | 31427789 | 2019 | Psychiatric | Frequency of consuming six or more units of alcohol | 0.01053 | 116394 |
| 3768 | 31427789 | 2019 | Psychiatric | Anxiety - Recent trouble relaxing | 0.01128 | 126422 |
| 4254 | 30952852 | 2019 | Psychiatric | Sleep duration (SD) | 0.01149 | 84441 |
| 3794 | 30804565 | 2019 | Psychiatric | Snoring | 0.01241 | 359916 |
| 8 | 22472876 | 2013 | Psychiatric | Major depressive disorder | 0.01260 | 18759 |
| 3228 | 31427789 | 2019 | Psychiatric | Sleep duration | 0.01270 | 384225 |
| 2024 | 23089632 | 2013 | Psychiatric | Alcohol dependence | 0.01311 | 2322 |
| 33 | 26362575 | 2016 | Psychiatric | Extraversion (IRT) | 0.01325 | 63661 |
| 3748 | 31427789 | 2019 | Psychiatric | Traumatic events - Felt loved as child | 0.01671 | 126348 |
| 1063 | 17728769 | 2007 | Psychiatric | Schizophrenia | 0.01722 | 2729 |
| 4315 | 30643251 | 2019 | Psychiatric | Cigarettes per day | 0.01765 | 263954 |
| 15 | 28439101 | 2017 | Psychiatric | Posttraumatic stress disorder | 0.01856 | 9223 |
| 3237 | 31427789 | 2019 | Psychiatric | Smoking/smokers in household | 0.01865 | 356053 |
| 4343 | 30531941 | 2018 | Psychiatric | Sleep sedentary | 0.02024 | 91105 |
| 4313 | 30643251 | 2019 | Psychiatric | Age of initiation of regular smoking | 0.02217 | 632802 |
| 3731 | 31427789 | 2019 | Psychiatric | Depression - Difficulty concentrating during worst ddepression | 0.02460 | 63297 |
| 3263 | 31427789 | 2019 | Psychiatric | Average weekly champagne plus white wine intake | 0.02965 | 273869 |
| 4327 | 30643256 | 2019 | Psychiatric | Well-being spectrum | 0.03074 | 2311184 |
| 2043 | 27329760 | 2016 | Psychiatric | Bipolar disorder | 0.03221 | 34950 |
| 25 | 24839885 | 2014 | Psychiatric | Internalizing | 0.03340 | 4596 |
| 4272 | 30846698 | 2019 | Psychiatric | Sleep duration | 0.03420 | 446118 |
| 2018 | 24369049 | 2014 | Psychiatric | Lithium response in Bipolar I patients - Alda Scale of 7 to 8 | 0.03646 | 294 |
| 1268 | 17903308 | 2007 | Psychiatric | Epworth Sleepiness Scale (FBAT) | 0.03915 | 721 |
| 4322 | 30643256 | 2019 | Psychiatric | Depressive symptoms (univariate) | 0.04590 | 1067913 |
| 4325 | 30643256 | 2019 | Psychiatric | Neuroticism (MA GWAMA) | 0.04770 | 523783 |
| 4316 | 30643251 | 2019 | Psychiatric | Smoking cessation | 0.04998 | 312821 |

**Supplementary Table 1B**. GWAS atlas output for *NT5DC1* for all psychiatric GWASs (n=321), retrieved on February 17^th^, 2020.

| **atlas ID** | **PMID** | **Year** | **Domain** | Trait | **P-value** | **N** |
| --- | --- | --- | --- | --- | --- | --- |
| **Bonferroni correction threshold** | | | | | **1.56E-04** |  |
| 4040 | 29906448 | 2018 | Psychiatric | Schizophrenia/Bipolar disorder | 8.54E-04 | 107620 |
| 4039 | 29906448 | 2018 | Psychiatric | Bipolar disorder | 0.00179 | 74194 |
| 3778 | 31427789 | 2019 | Psychiatric | Traumatic events - Victim of physically violent crime | 0.00703 | 126580 |
| 3725 | 31427789 | 2019 | Psychiatric | Frequency of drinking alcohol | 0.01240 | 126656 |
| 1064 | 19488044 | 2009 | Psychiatric | Bipolar disorder | 0.01509 | 2035 |
| 4274 | 30846698 | 2019 | Psychiatric | Short sleep | 0.02691 | 411934 |
| 8 | 22472876 | 2013 | Psychiatric | Major depressive disorder | 0.03234 | 18759 |
| 3239 | 31427789 | 2019 | Psychiatric | Exposure to tobacco smoke outside home | 0.03400 | 327794 |

**Supplementary Table 1C.** GWAS atlas output for *NT5DC2* for all psychiatric GWASs (n=321), retrieved on February 17^th^, 2020.

| **atlas ID** | **PMID** | **Year** | **Domain** | **Trait** | **P-value** | **N** |
| --- | --- | --- | --- | --- | --- | --- |
| 3798 | 29942085 | 2018 | Psychiatric | Worry subcluster | 1.84E-11 | 348219 |
| 3997 | 29500382 | 2018 | Psychiatric | Worrier / anxious feelings (WORRY) | 7.14E-11 | 264646 |
| 3290 | 31427789 | 2019 | Psychiatric | Worrier / anxious feelings | 1.25E-10 | 376411 |
| 4040 | 29906448 | 2018 | Psychiatric | Schizophrenia/Bipolar disorder | 8.49E-09 | 107620 |
| 4327 | 30643256 | 2019 | Psychiatric | Well-being spectrum | 1.74E-08 | 2311184 |
| 3982 | 29483656 | 2018 | Psychiatric | Schizophrenia | 3.08E-08 | 105318 |
| 11 | 25056061 | 2014 | Psychiatric | Schizophrenia | 5.71E-08 | 82315 |
| 13 | 24280982 | 2014 | Psychiatric | Schizophrenia vs Bipolar disorder | 5.71E-08 | 16381 |
| 3996 | 29500382 | 2018 | Psychiatric | Nervous feelings (NERV-FEEL) | 1.12E-07 | 264858 |
| 3289 | 31427789 | 2019 | Psychiatric | Nervous feelings | 2.09E-07 | 376368 |
| 4325 | 30643256 | 2019 | Psychiatric | Neuroticism (MA GWAMA) | 3.53E-07 | 523783 |
| 1191 | 23453885 | 2013 | Psychiatric | PGC cross disorder | 1.025E-06 | 61220 |
| 3795 | 29942085 | 2018 | Psychiatric | Neuroticism | 1.115E-06 | 390278 |
| 4255 | 30952852 | 2019 | Psychiatric | Sleep efficiency | 1.738E-06 | 84810 |
| 4326 | 30643256 | 2019 | Psychiatric | Depressive symptoms (MA GWAMA) | 1.935E-06 | 1067913 |
| 4321 | 30643256 | 2019 | Psychiatric | Neuroticism (univariate) | 4.117E-06 | 523783 |
| 3990 | 29500382 | 2018 | Psychiatric | Neuroticism sum score | 4.413E-06 | 380506 |
| 4322 | 30643256 | 2019 | Psychiatric | Depressive symptoms (univariate) | 6.223E-06 | 1067913 |
| 3728 | 31427789 | 2019 | Psychiatric | Anxiety - Ever worried more than most people would in similar situation | 1.293E-05 | 106215 |
| 3231 | 31427789 | 2019 | Psychiatric | Nap during day | 1.315E-05 | 386124 |
| 3417 | 31427789 | 2019 | Psychiatric | Neuroticism score | 1.79E-05 | 312740 |
| 4038 | 29906448 | 2018 | Psychiatric | Schizophrenia | 1.874E-05 | 87491 |
| 4000 | 29500382 | 2018 | Psychiatric | Suffer from 'nerves' (SUF-NERV) | 8.999E-05 | 262321 |
| 3291 | 31427789 | 2019 | Psychiatric | Tense / 'highly strung' | 9.916E-05 | 374129 |
| 3998 | 29500382 | 2018 | Psychiatric | Tense / 'highly strung' (TENSE) | 0.000115 | 263635 |
| 3299 | 31427789 | 2019 | Psychiatric | Frequency of tenseness / restlessness in last 2 weeks | 0.000125 | 371869 |
| 4249 | 30952852 | 2019 | Psychiatric | Diurnal inactivity duration | 0.000150 | 84757 |
| **Bonferroni correction threshold** | | | | | **1.56E-04** |  |
|  |  |  |  |  |  |  |
| 4039 | 29906448 | 2018 | Psychiatric | Bipolar disorder | 0.000156 | 74194 |
| 3745 | 31427789 | 2019 | Psychiatric | Happiness and subjective well-being - General happiness | 0.000167 | 126132 |
| 3293 | 31427789 | 2019 | Psychiatric | Suffer from 'nerves' | 0.000238 | 372273 |
| 4337 | 30531941 | 2018 | Psychiatric | Sleep durataion | 0.000253 | 91105 |
| 4293 | 30718901 | 2019 | Psychiatric | Depression | 0.000340 | 500199 |
| 4338 | 30531941 | 2018 | Psychiatric | Sleep durataion (conditioning sex and BMI) | 0.000361 | 91105 |
| 4270 | 30867560 | 2019 | Psychiatric | Anxiety/tension factors | 0.000448 | 270059 |
| 4271 | 30867560 | 2019 | Psychiatric | Worry/vulnerability factors | 0.000448 | 270059 |
| 10 | 23974872 | 2013 | Psychiatric | Schizophrenia | 0.000500 | 32143 |
| 9 | 21926974 | 2011 | Psychiatric | Schizophrenia | 0.000508 | 21856 |
| 3302 | 31427789 | 2019 | Psychiatric | Seen a psychiatrist for nerves, anxiety, tension or depression | 0.000562 | 384700 |
| 2043 | 27329760 | 2016 | Psychiatric | Bipolar disorder | 0.000882 | 34950 |
| 4002 | 29500382 | 2018 | Psychiatric | Guilty feelings (GUILT) | 0.000937 | 265139 |
| 4011 | 29662059 | 2018 | Psychiatric | Broad depression | 0.001133 | 322580 |
| 12 | 24280982 | 2014 | Psychiatric | Schizophrenia/Bipolar disorder | 0.001368 | 39202 |
| 3762 | 31427789 | 2019 | Psychiatric | Anxiety - Recent inability to stop or control worying | 0.001373 | 126300 |
| 3295 | 31427789 | 2019 | Psychiatric | Guilty feelings | 0.001452 | 376361 |
| 3995 | 29500382 | 2018 | Psychiatric | Fed-up feelings (FED_UP) | 0.001463 | 266208 |
| 3288 | 31427789 | 2019 | Psychiatric | Fed-up feelings | 0.001519 | 378357 |
| 4037 | BioRxiv: https://doi.org/10.1101/224774 | 2017 | Psychiatric | Autism spectrum disorder | 0.001549 | 46350 |
| 3300 | 31427789 | 2019 | Psychiatric | Frequency of tiredness / lethargy in last 2 weeks | 0.001960 | 375053 |
| 4315 | 30643251 | 2019 | Psychiatric | Cigarettes per day | 0.002014 | 263954 |
| 3284 | 31427789 | 2019 | Psychiatric | Mood swings | 0.002125 | 377179 |
| 3991 | 29500382 | 2018 | Psychiatric | Mood swings (MOOD) | 0.002509 | 265382 |
| 3724 | 31427789 | 2019 | Psychiatric | Frequency of inability to cease drinking in last year | 0.002520 | 69164 |
| 3425 | 31427789 | 2019 | Psychiatric | Ever smoked | 0.003348 | 385013 |
| 3301 | 31427789 | 2019 | Psychiatric | Seen doctor (GP) for nerves, anxiety, tension or depression | 0.003501 | 383771 |
| 4252 | 30952852 | 2019 | Psychiatric | Number of sleep episodes | 0.003533 | 84810 |
| 3656 | 31427789 | 2019 | Psychiatric | Alcohol - Alcohol drinker status: Never | 0.004141 | 386082 |
| 4274 | 30846698 | 2019 | Psychiatric | Short sleep | 0.005171 | 411934 |
| 5 | 21926972 | 2011 | Psychiatric | Bipolar disorder | 0.009589 | 16731 |
| 4273 | 30846698 | 2019 | Psychiatric | Long sleep | 0.011258 | 339926 |
| 26 | 26087016 | 2016 | Psychiatric | Agression | 0.011728 | 18988 |
| 4314 | 30643251 | 2019 | Psychiatric | Ever smoked regulary | 0.012829 | 262990 |
| 3797 | 29942085 | 2018 | Psychiatric | Depressive affect subcluster | 0.012943 | 357957 |
| 4014 | 29700475 | 2018 | Psychiatric | Major depressive disorder | 0.014443 | 173005 |
| 55 | 27089181 | 2016 | Psychiatric | Neuroticism | 0.014901 | 170911 |
| 3761 | 31427789 | 2019 | Psychiatric | Depression - Recent trouble concentrating on things | 0.017330 | 126633 |
| 4 | 28540026 | 2017 | Psychiatric | Autism spectrum disorder | 0.018494 | 15954 |
| 3765 | 31427789 | 2019 | Psychiatric | Anxiety - Recent feelings of foreboding | 0.019967 | 126179 |
| 4087 | 29255261 | 2018 | Psychiatric | Neuroticism | 0.020574 | 329821 |
| 3604 | 31427789 | 2019 | Psychiatric | Non-cancer illness code, self-reported: depression | 0.021881 | 289307 |
| 4170 | 29970889 | 2018 | Psychiatric | Loneliness (MTAG) | 0.023981 | 487647 |
| 3511 | 31427789 | 2019 | Psychiatric | Reason for reducing amount of alcohol drunk: Other reason | 0.025009 | 142645 |
| 3509 | 31427789 | 2019 | Psychiatric | Reason for reducing amount of alcohol drunk: Illness or ill health | 0.030149 | 142645 |
| 1063 | 17728769 | 2007 | Psychiatric | Schizophrenia | 0.030480 | 2729 |
| 4269 | 30867560 | 2019 | Psychiatric | Neuroticism general factor | 0.033494 | 270059 |
| 3385 | 31427789 | 2019 | Psychiatric | Happiness | 0.035037 | 128677 |
| 7 | 26754954 | 2016 | Psychiatric | Anxiety disorder (factor score) | 0.036644 | 18186 |
| 3335 | 31427789 | 2019 | Psychiatric | Light smokers, at least 100 smokes in lifetime | 0.039497 | 105610 |
| 30 | 21173776 | 2012 | Psychiatric | Agreablenes (NEO-FFI) | 0.039962 | 17375 |
| 4317 | 30643251 | 2019 | Psychiatric | Drinks per day | 0.040595 | 537349 |
| 3792 | 30804565 | 2019 | Psychiatric | Daytime napping | 0.042943 | 386577 |
| 3749 | 31427789 | 2019 | Psychiatric | Traumatic events - Someone to take to doctor when needed as a child | 0.049922 | 125985 |

**Supplementary Table 1D.** GWAS atlas output for *NT5DC3* for all psychiatric GWASs (n=321), retrieved on February 17^th^, 2020.

| **atlas ID** | **PMID** | **Year** | **Domain** | **Trait** | **P-value** | **N** |
| --- | --- | --- | --- | --- | --- | --- |
| **Bonferroni correction threshold** | | | | | **1.56E-04** |  |
| 3264 | 31427789 | 2019 | Psychiatric | Average weekly beer plus cider intake | 4.22E-04 | 274556 |
| 4249 | 30952852 | 2019 | Psychiatric | Diurnal inactivity duration | 6.39E-04 | 84757 |
| 3727 | 31427789 | 2019 | Psychiatric | Anxiety - Ever felt worried, tense, or anxious for most of a month or longer | 0.00157 | 118397 |
| 2025 | 22952603 | 2012 | Psychiatric | 10 mg response to amphetamine | 0.00190 | 381 |
| 3239 | 31427789 | 2019 | Psychiatric | Exposure to tobacco smoke outside home | 0.00244 | 327794 |
| 3729 | 31427789 | 2019 | Psychiatric | Depression - Age at first episode of depression | 0.00335 | 65776 |
| 3982 | 29483656 | 2018 | Psychiatric | Schizophrenia | 0.00380 | 105318 |
| 11 | 25056061 | 2014 | Psychiatric | Schizophrenia | 0.00765 | 82315 |
| 13 | 24280982 | 2014 | Psychiatric | Schizophrenia vs Bipolar disorder | 0.00765 | 16381 |
| 2026 | 22952603 | 2012 | Psychiatric | Baseline positive affect factor score | 0.01054 | 381 |
| 4038 | 29906448 | 2018 | Psychiatric | Schizophrenia | 0.02138 | 87491 |
| 3355 | 31427789 | 2019 | Psychiatric | Ever stopped smoking for 6+ months | 0.02193 | 93871 |

**Supplementary Table 1E.** GWAS atlas output for *NT5DC4* for all psychiatric GWASs (n=321), retrieved on February 17^th^, 2020.

| **atlas ID** | **PMID** | **Year** | **Domain** | **Trait** | **P-value** | **N** |
| --- | --- | --- | --- | --- | --- | --- |
| **Bonferroni correction threshold** | | | | | **1.56E-04** |  |
| 4000 | 29500382 | 2018 | Psychiatric | Suffer from 'nerves' (SUF-NERV) | 2.80E-04 | 262321 |
| 3293 | 31427789 | 2019 | Psychiatric | Suffer from 'nerves' | 9.41E-04 | 372273 |
| 1217 | 27911795 | 2016 | Psychiatric | Alcohol consumption (dichotomous, male) | 0.00195 | 26991 |
| 4273 | 30846698 | 2019 | Psychiatric | Long sleep | 0.00236 | 339926 |
| 4272 | 30846698 | 2019 | Psychiatric | Sleep duration | 0.00319 | 446118 |
| 1216 | 27911795 | 2016 | Psychiatric | Alcohol consumption (dichotomous) | 0.00522 | 74711 |
| 4250 | 30952852 | 2019 | Psychiatric | L5 timing | 0.00552 | 85205 |
| 3229 | 31427789 | 2019 | Psychiatric | Getting up in morning | 0.01022 | 385494 |
| 3287 | 31427789 | 2019 | Psychiatric | Sensitivity / hurt feelings | 0.01049 | 375272 |
| 3798 | 29942085 | 2018 | Psychiatric | Worry subcluster | 0.01106 | 348219 |
| 3416 | 31427789 | 2019 | Psychiatric | Bipolar and major depression status | 0.01230 | 93296 |
| 3994 | 29500382 | 2018 | Psychiatric | Sensitivity / hurt feelings (HURT) | 0.01230 | 264144 |
| 3228 | 31427789 | 2019 | Psychiatric | Sleep duration | 0.01291 | 384225 |
| 3997 | 29500382 | 2018 | Psychiatric | Worrier / anxious feelings (WORRY) | 0.01696 | 264646 |
| 3791 | 30804565 | 2019 | Psychiatric | Ease of getting up in the morning | 0.01910 | 385949 |
| 3740 | 31427789 | 2019 | Psychiatric | Depression - Depression possibly related to stressful or traumatic event | 0.02292 | 71568 |
| 3753 | 31427789 | 2019 | Psychiatric | Traumatic events - Repeated disturbing thoughts of stressful experience in past month | 0.02345 | 126635 |
| 2054 | 23377640 | 2013 | Psychiatric | Percentage improvement after 12 weeks of antidepressant treatment in MDD | 0.03137 | 2256 |

**Supplementary Table 2.** Overview of primer sequences used in this paper.

| **Primer name** | **F/R** | **Reference** | **Sequence** |
| --- | --- | --- | --- |
| C_Genomic | F | [1] | 5’-GCCCACTGTCAGCTCTCAAC-3’ |
| NC_Genomic | F |  | 5’-GCTGGCGAACTGTCAATCAC-3’ |
| pKC26 | R |  | 5’-TGTAAAACGACGGCCAGT-3’ |
| eIF-1A | F | This study | 5’-ATCAGCTCCGAGGATGACGC-3’ |
|  | R |  | 5’-GCCGAGACAGACGTTCCAGA-3’ |
| αTub84B | F |  | 5’- TGTCGCGTGTGAAACACTTC-3’ |
|  | R |  | 5’- AGCAGGCGTTTCCAATCTG-3’ |
| CG1814 (dNT5C) | F |  | 5’-CTGCTGCCTATTGCCTACG-3’ |
|  | R |  | 5’-CTGCTGCCTATTGCCTACG-3’ |
| CG32549 (dNT5B) | F |  | 5’-AACAGATCGCTGCATTTGG-3’ |
|  | R |  | 5’-CTCCTTGACCAGGTTGAAGC-3’ |
| CG2277 (dNT5C) | F | #PP20616 [6] | 5’-CGTCTCCTGTTACGCAATCTC-3’ |
|  | R |  | 5’-CGTCACTTCTCGTGTTGCTCT-3’ |
| dNT5A RNAi 1&2 | F | This study | 5’-CTGCCCTTTCACTTCGACTG-3’ |
|  | R |  | 5’-CGCCAATTTATCGAGATTGC-3’ |
| dNT5B RNAi 1&2 | F |  | 5’-TGCGCAGTGTGTTATGAAATC-3’ |
|  | R |  | 5’-TTCCAGCCACTTGATTTTCC-3’ |
| dNT5C RNAi 1&2 | F |  | 5’-AGGTGCAGGTGTACGGATTC-3’ |
|  | R |  | 5’-CGAGCCAGATTGTACAGCAG-3’ |

**Supplementary Table 3.** Summary statistics of pan-neuronal knockdown of *dNT5* genes in the habituation assay.

| **Genotype** | **n total** | **n jumpers** | **TTC** | |
| --- | --- | --- | --- | --- |
|  |  |  | **Mean** | **SD** |
| ***w*; 2x*GMR*-*wIR*; *nSyb*-*Gal4*, *UAS*-*Dcr2*/+** | 192 | 112 | 10.075 | 4.0165 |
| ***w*; 2x*GMR*-*wIR*; *nSyb*-*Gal4*, *UAS*-*Dcr2*/*UAS*-*dNT5A* *RNAi_1_*** | 192 | 122 | 9.9124 | 6.7999 |
| ***w*; 2x*GMR*-*wIR*; *nSyb*-*Gal4*, *UAS*-*Dcr2*/*UAS*-*dNT5B* *RNAi*** | 176 | 97 | 6.8601 | 3.935 |
| ***w*; 2x*GMR*-*wIR*; *nSyb*-*Gal4*, *UAS*-*Dcr2*/*UAS*-*dNT5C* *RNAi_1_*** | 192 | 161 | 21.1875*** | 10.61 |
|  | | | | |
| ***w*; 2x*GMR*-*wIR*/+; *nSyb*-*Gal4*, *UAS*-*Dcr2*** | 160 | 123 | 15.423 | 4.5447 |
| ***w*; 2x*GMR*-*wIR*/*UAS*-*dNT5A* *RNAi_2_*; *nSyb*-*Gal4*, *UAS*-*Dcr2*** | 192 | 176 | 46.0791*** | 11.16 |
| ***w*; 2x*GMR*-*wIR*/*UAS*-*dNT5C* *RNAi_2_*; *nSyb*-*Gal4*, *UAS*-*Dcr2*** | 160 | 138 | 34.7556*** | 11.079 |

*p_adj_<0.05, **p_adj_<0.01, ***p_adj_<0.001

**Supplementary Table 4A.** Summary statistics of activity and sleep parameters from knockdown with RNAi_1_ and genetic background control.

|  |  | ***w*, *UAS*-*Dcr2*;; *nSyb*-*Gal4*/+**  **(n=88)** | | ***w*, *UAS*-*Dcr2*;; *nSyb*-*Gal4*/*UAS*-*dNT5A* *RNAi_1_***  **(n=84)** | | ***w*, *UAS*-*Dcr2*;; *nSyb*-*Gal4*/*UAS*-*dNT5B* *RNAi***  **(n=71)** | | ***w*, *UAS*-*Dcr2*;; *nSyb*-*Gal4*/*UAS*-*dNT5C* *RNAi_1_***  **(n=86)** | |
| --- | --- | --- | --- | --- | --- | --- | --- | --- | --- |
|  |  | **Mean** | **SD** | **Mean** | **SD** | **Mean** | **SD** | **Mean** | **SD** |
| **Total activity** | **Day** | 494.4 | 252.9 | 350.6*** | 171 | 486.6 | 287.5 | 442.4 | 285.9 |
|  | **Night** | 611 | 249.5 | 641.2 | 260.5 | 520.9 | 196.9 | 491** | 255.9 |
| **Total sleep** | **Day** | 536.6 | 70 | 584.7*** | 60.95 | 551.4 | 79.21 | 542.4 | 79.82 |
|  | **Night** | 495.7 | 87.83 | 507.2 | 81.01 | 550.0*** | 66.91 | 528.7* | 90.32 |
| **Activity while awake** | **Day** | 2.585 | 0.5701 | 2.517 | 0.6054 | 2.825* | 0.6785 | 2.329* | 0.672 |
|  | **Night** | 2.747 | 0.5239 | 3.01** | 0.4109 | 3.134*** | 0.5689 | 2.551* | 0.4854 |
| **Sleep latency** | **Day** | 57.92 | 27.95 | 44.12** | 23.84 | 45* | 32.12 | 62 | 37.19 |
|  | **Night** | 56.79 | 36.99 | 67.79 | 41.24 | 45.52 | 35.55 | 51.88 | 35.2 |
| **Sleep bout** | **Day** | 11.92 | 3.978 | 8.94*** | 3.253 | 11.27 | 4.379 | 9.506*** | 4.331 |
|  | **Night** | 9.929 | 3.344 | 10.7 | 4.411 | 11.67* | 4.512 | 9.305 | 3.791 |
| **Sleep bout duration** | **Day** | 65.63 | 31.75 | 86.63* | 37.85 | 66.34 | 43.67 | 96.79*** | 73.91 |
|  | **Night** | 63.96 | 27.23 | 64.67 | 31.66 | 62.73 | 35.68 | 86.72** | 63.75 |

*p_adj_<0.05, **p_adj_<0.01, ***p_adj_<0.001

**Supplementary Table 4B.** Summary statistics of activity and sleep parameters from knockdown with RNAi_2_ and genetic background control.

|  |  | ***w*, *UAS*-*Dcr2*; +; *nSyb*-*Gal4***  **(n=62)** | | ***w*, *UAS*-*Dcr2*; *UAS*-*dNT5A* *RNAi_2_*; *nSyb*-*Gal4***  **(n=77)** | | ***w*, *UAS*-*Dcr2*; *UAS*-*dNT5C* *RNAi_2_*; *nSyb*-*Gal4***  **(n=61)** | |
| --- | --- | --- | --- | --- | --- | --- | --- |
|  |  | **Mean** | **SD** | **Mean** | **SD** | **Mean** | **SD** |
| **Total activity** | **Day** | 444.9 | 244.3 | 582.9** | 228.1 | 469.4 | 221.2 |
|  | **Night** | 451.6 | 185.6 | 643.8** | 418.1 | 527.6 | 276 |
| **Total sleep** | **Day** | 538.9 | 54.01 | 510.7* | 67.65 | 530.6 | 56.77 |
|  | **Night** | 551.6 | 75.72 | 507.5* | 111.7 | 516.9 | 100.9 |
| **Activity while awake** | **Day** | 2.376 | 0.7843 | 2.753** | 0.5792 | 2.461 | 0.6426 |
|  | **Night** | 2.83 | 0.7797 | 3.006 | 0.6417 | 2.659 | 0.543 |
| **Sleep latency** | **Day** | 53.02 | 24.92 | 75.69*** | 34.11 | 70.69** | 33.89 |
|  | **Night** | 44.16 | 24.67 | 54.95 | 56.48 | 51.92 | 37.25 |
| **Sleep bout** | **Day** | 12.71 | 4.046 | 11.65 | 3.835 | 12.87 | 4.569 |
|  | **Night** | 10.25 | 3.558 | 10.72 | 4.748 | 10.17 | 5.617 |
| **Sleep bout duration** | **Day** | 51.73 | 19.37 | 57.63 | 25.3 | 51.63 | 20.4 |
|  | **Night** | 75.13 | 46.32 | 72.7 | 44.62 | 91.85 | 89.36 |

*p_adj_<0.05, **p_adj_<0.01, ***p_adj_<0.001

**Supplementary Table 5A.** Summary statistics of NMJ synapse parameters from knockdown with RNAi_1_ and genetic background control.

|  | ***w*, *UAS*-*Dcr2*;; *nSyb*-*Gal4*/+**  **(n=36)** | | ***w*, *UAS*-*Dcr2*;; *nSyb*-*Gal4*/*UAS*-*dNT5A* *RNAi_1_***  **(n=34)** | | ***w*, *UAS*-*Dcr2*;; *nSyb*-*Gal4*/*UAS*-*dNT5B* *RNAi***  **(n=29)** | | ***w*, *UAS*-*Dcr2*;; *nSyb*-*Gal4*/*UAS*-*dNT5C* *RNAi_1_***  **(n=15)** | |
| --- | --- | --- | --- | --- | --- | --- | --- | --- |
|  | **Mean** | **SD** | **Mean** | **SD** | **Mean** | **SD** | **Mean** | **SD** |
| **Area** | 408.6 | 49.46 | 393.4 | 76.19 | 386 | 70.7 | 447.2 | 61.31 |
| **Perimeter** | 384.6 | 78.18 | 340.3 | 81.4 | 340 | 78.59 | 390.4 | 95.8 |
| **Length** | 119.5 | 17.69 | 109.1 | 24.37 | 116 | 22.29 | 123.8 | 14.11 |
| **Longest branch length** | 107.6 | 21.52 | 98.44 | 23.78 | 103.9 | 24.49 | 109.2 | 19.95 |
| **Bouton** | 22.42 | 3.367 | 19.62** | 4.178 | 20.86 | 3.378 | 22 | 2.563 |
| **Active zone** | 342.2 | 63.82 | 303.8 | 89.14 | 351.2 | 110.5 | 339.3 | 57.05 |
| **Branch** | 4.889 | 2.175 | 4.588 | 2.463 | 5.138 | 2.85 | 4.667 | 1.676 |
| **Branching point** | 1.444 | 1.182 | 1.382 | 1.129 | 1.621 | 1.374 | 1.467 | 0.9904 |
| **Island** | 2 | 0.7928 | 1.824 | 0.8338 | 1.897 | 0.772 | 1.733 | 0.7988 |

*p_adj_<0.05, **p_adj_<0.01, ***p_adj_<0.001

**Supplementary Table 5B.** Summary statistics of NMJ synapse parameters from knockdown with RNAi_2_ and genetic background control.

|  | ***w*, *UAS*-*Dcr2*; +; *nSyb*-*Gal4***  **(n=40)** | | ***w*, *UAS*-*Dcr2*; *UAS*-*dNT5A* *RNAi_2_*; *nSyb*-*Gal4***  **(n=30)** | | ***w*, *UAS*-*Dcr2*; *UAS*-*dNT5C* *RNAi_2_*; *nSyb*-*Gal4***  **(n=26)** | |
| --- | --- | --- | --- | --- | --- | --- |
|  | **Mean** | **SD** | **Mean** | **SD** | **Mean** | **SD** |
| **Area** | 330.2 | 63.11 | 343.7 | 78.93 | 353.6 | 82.92 |
| **Perimeter** | 253.3 | 62.52 | 258.8 | 58.96 | 299.2* | 90.75 |
| **Length** | 101.9 | 20.88 | 108.4 | 25.8 | 110.4 | 27.61 |
| **Longest branch length** | 90.09 | 22.19 | 98.82 | 23.95 | 101.5 | 27.64 |
| **Bouton** | 19.33 | 3.931 | 18.77 | 4.688 | 18.92 | 5.098 |
| **Active zone** | 272.5 | 65.28 | 272.4 | 76 | 230.8 | 68.95 |
| **Branch** | 4.725 | 2.298 | 4.6 | 2.401 | 4.308 | 2.055 |
| **Branching point** | 1.45 | 1.218 | 1.433 | 1.194 | 1.115 | 0.9931 |
| **Island** | 1.825 | 0.7472 | 1.733 | 0.9072 | 2.077 | 1.055 |

*p_adj_<0.05, **p_adj_<0.01, ***p_adj_<0.001

1. Green, E.W., et al., *A Drosophila RNAi collection is subject to dominant phenotypic effects.* Nat Methods, 2014. **11**(3): p. 222-3.

2. Vissers, J.H., et al., *A Drosophila RNAi library modulates Hippo pathway-dependent tissue growth.* Nat Commun, 2016. **7**: p. 10368.

3. Untergasser, A., et al., *Primer3--new capabilities and interfaces.* Nucleic Acids Res, 2012. **40**(15): p. e115.

4. Pfaffl, M.W., *A new mathematical model for relative quantification in real-time RT-PCR.* Nucleic Acids Res, 2001. **29**(9): p. e45.

5. Sievers, F. and D.G. Higgins, *Clustal Omega for making accurate alignments of many protein sequences.* Protein Sci, 2018. **27**(1): p. 135-145.

6. Hu, Y., et al., *FlyPrimerBank: an online database for Drosophila melanogaster gene expression analysis and knockdown evaluation of RNAi reagents.* G3 (Bethesda), 2013. **3**(9): p. 1607-16.
