# Supplementary figures and images for: Investigating cytosolic 5′-nucleotidase II family genes as candidates for neuropsychiatric disorders in Drosophila (114/150 chr)
Source: Transl Psychiatry. 2021 Jan 18;11:55. doi: 10.1038/s41398-020-01149-x (PMC7813868; doi:10.1038/s41398-020-01149-x)

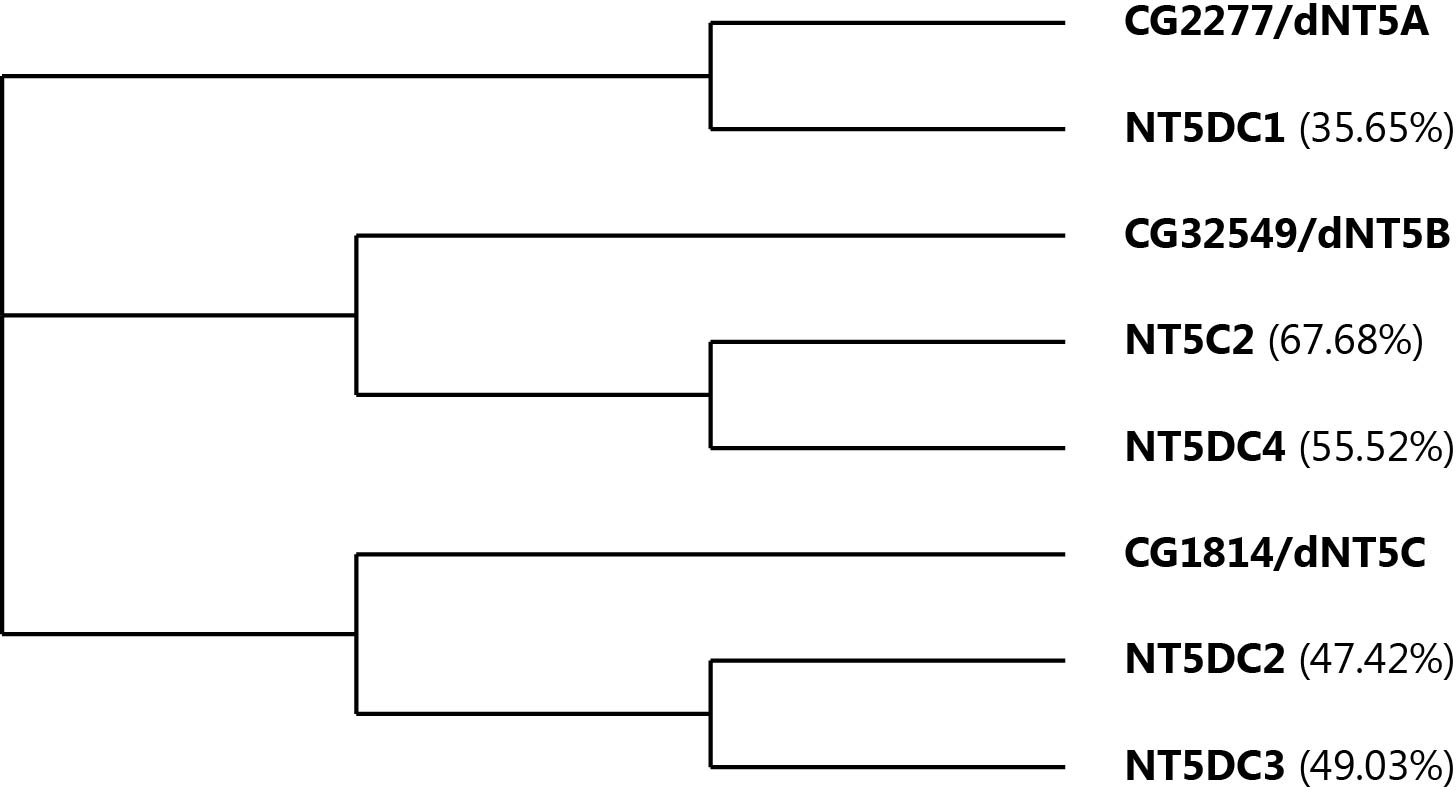

Supplement: Supplementary file 2 — Supplementary Figure 1 [file 41398_2020_1149_MOESM2_ESM.jpg]

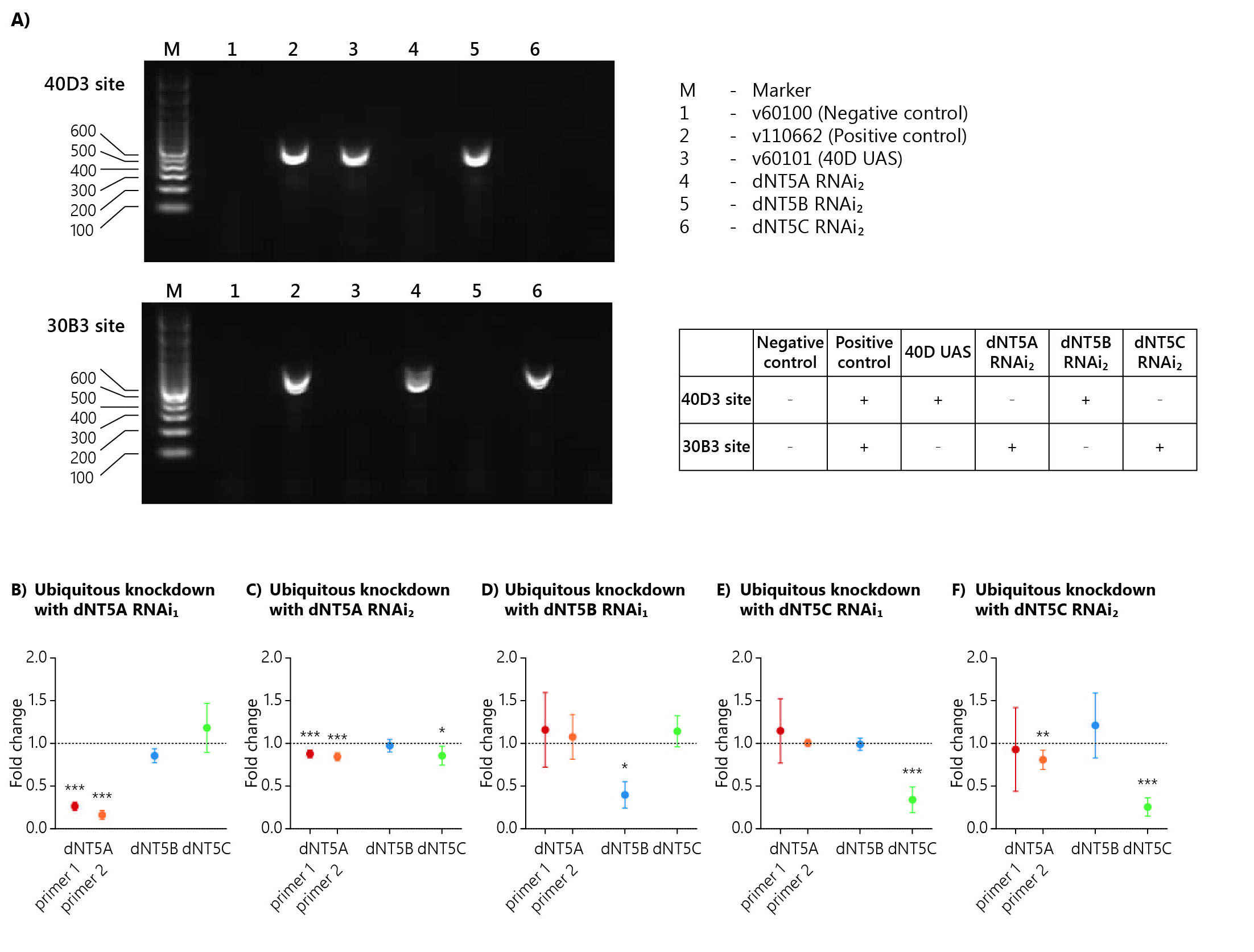

Supplement: Supplementary file 3 — Supplementary Figure 2 [file 41398_2020_1149_MOESM3_ESM.jpg]

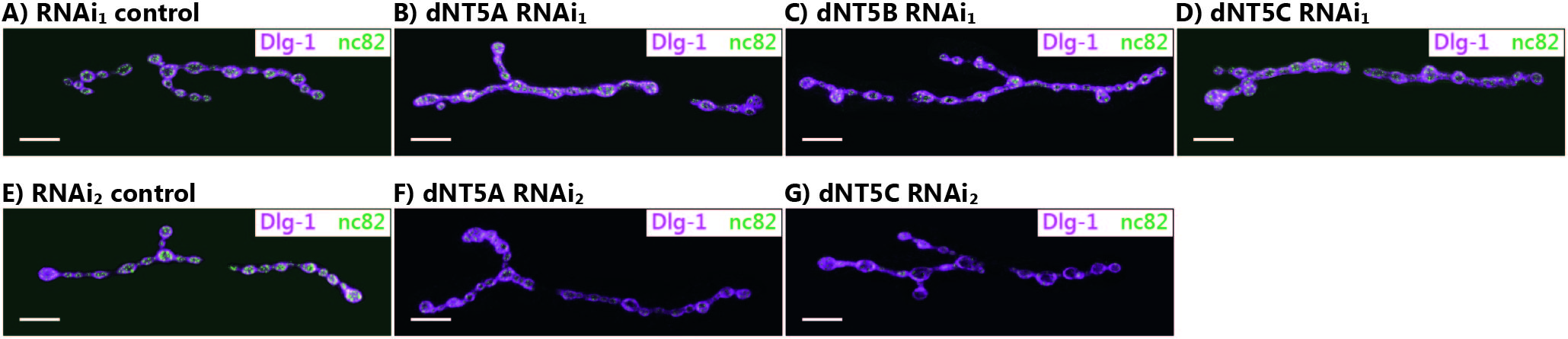

Supplement: Supplementary file 4 — Supplementary Figure 3 [file 41398_2020_1149_MOESM4_ESM.jpg]
